# The Arabidopsis Cysteine-Rich Receptor-Like Kinase CRK36 Regulates Immunity through Interaction with the Cytoplasmic Kinase BIK1

**DOI:** 10.3389/fpls.2017.01856

**Published:** 2017-10-27

**Authors:** Dong Sook Lee, Young Cheon Kim, Sun Jae Kwon, Choong-Min Ryu, Ohkmae K. Park

**Affiliations:** ^1^Department of Life Sciences, Korea University, Seoul, South Korea; ^2^Molecular Phytobacteriology Laboratory, KRIBB, Daejeon, South Korea

**Keywords:** *Arabidopsis thaliana*, pattern-triggered immunity, stomatal immunity, cysteine-rich receptor-like kinase, CRK36, BiK1, FLS2, NADPH oxidase

## Abstract

Receptor-like kinases are important signaling components that regulate a variety of cellular processes. In this study, an Arabidopsis cDNA microarray analysis led to the identification of the cysteine-rich receptor-like kinase CRK36 responsive to the necrotrophic fungal pathogen, *Alternaria brassicicola*. To determine the function of *CRK36* in plant immunity, T-DNA-insertion knockdown (*crk36*) and overexpressing (*CRK36OE*) plants were prepared. *CRK36OE* plants exhibited increased hypersensitive cell death and ROS burst in response to avirulent pathogens. Treatment with a typical pathogen-associated molecular pattern, flg22, markedly induced pattern-triggered immune responses, notably stomatal defense, in *CRK36OE* plants. The immune responses were weakened in *crk36* plants. Protein-protein interaction assays revealed the *in vivo* association of CRK36, FLS2, and BIK1. CRK36 enhanced flg22-triggered BIK1 phosphorylation, which showed defects with Cys mutations in the DUF26 motifs of CRK36. Disruption of *BIK1* and *RbohD/RbohF* genes further impaired CRK36-mediated stomatal defense. We propose that CRK36, together with BIK1 and NADPH oxidases, may form a positive activation loop that enhances ROS burst and leads to the promotion of stomatal immunity.

## Introduction

Plants are exposed to a myriad of microbes during their lifespan and they have evolved two layers of immunity to defend against microbial pathogens. The first layer of immunity is referred to as pattern-triggered immunity (PTI), which is triggered upon recognition of pathogen-associated molecular patterns (PAMPs) by pattern recognition receptors (PRRs) (Macho and Zipfel, 2014; Zipfel, 2014). Activation of PRRs by PAMPs triggers PTI responses, including production of reactive oxygen species (ROS), ion fluxes, activation of mitogen activated protein kinases (MAPKs), expression of defense-related genes, callose deposition, and stomatal closure (Boller and Felix, 2009; Nicaise et al., 2009). One of the best characterized PRRs is the leucine-rich repeat (LRR) receptor-like kinase (RLK) flagellin-sensing 2 (FLS2), which recognizes flagellin or flagellin-derived peptide flg22 (Chinchilla et al., 2006; Robatzek et al., 2006). Upon ligand binding, FLS2 recruits another LRR-RLK brassinosteroid insensitive 1 (BRI1)-associated kinase 1 (BAK1), forming an active receptor complex (Chinchilla et al., 2007; Heese et al., 2007; Monaghan and Zipfel, 2012; Koller and Bent, 2014). This leads to phosphorylation and activation of the downstream receptor-like cytoplasmic kinases (RLCKs), *Botrytis*-induced kinase (BIK1), and other PBS1-like (PBL) proteins (Veronese et al., 2006; Lu et al., 2010; Zhang et al., 2010). Activated BIK1 dissociates from the receptors, and in turn, phosphorylates the NADPH oxidase RbohD, leading to ROS burst and stomatal immunity (Kadota et al., 2014; Li et al., 2014).

PTI can be overcome by pathogenic microbes that inject effectors into plant cells through the type III secretion system, resulting in effector-triggered susceptibility (ETS) (Shao et al., 2003; Zhang et al., 2007; Shan et al., 2008). As a result of coevolution, plants have developed a second layer of plant immunity, the so-called effector-triggered immunity (ETI), in which plant resistance (R) proteins lead to the recognition of effectors and suppression of ETS (Mackey et al., 2002). ETI induces stronger and more prolonged immune responses than PTI and is frequently associated with programmed cell death (PCD), referred to as the hypersensitive response (HR), which restricts pathogen growth at the infection site (Coll et al., 2011). ETI and PTI activate common responses, including transcriptional reprogramming, ROS production, and MAPK activation, which suggests that they share downstream signaling components (Tsuda and Katagiri, 2010).

RLKs are conserved upstream signaling molecules that regulate a number of biological processes, including plant development and stress adaptation (Diévart and Clark, 2004; Osakabe et al., 2013). RLKs belong to one of the largest gene families with more than 600 members in Arabidopsis, and are classified into subfamilies according to the types of their extracellular domains (Shiu and Bleecker, 2001). Cysteine-rich receptor-like kinases (CRKs), also called domain of unknown function 26 (DUF26) RLKs, form a subfamily of RLKs with more than 40 members (Chen, 2001; Wrzaczek et al., 2010). Their extracellular domains contain 2 copies of a DUF26 motif having the sequence C-X8-C-X2-C with conserved Cys residues. CRKs play roles in disease resistance and cell death in plants (Chen et al., 2003; Acharya et al., 2007; Ederli et al., 2011). In gene expression analyses, *CRKs* were induced by treatments with O_3_, salicylic acid (SA), PAMPs, and pathogens (Du and Chen, 2000; Wrzaczek et al., 2010). Overexpression of *CRK5* and *CRK13* in Arabidopsis led to enhanced resistance to *Pseudomonas syringae* pv. tomato (*Pst*) DC3000 and HR-like cell death (Chen et al., 2003; Acharya et al., 2007). CRK4, CRK19, and CRK20 also activated rapid cell death in transgenic plants (Chen et al., 2004). *CRK45* (also called *ACRK1*)-overexpressing plants showed enhanced resistance to *Pst* DC3000, whereas *crk45* mutant plants were more susceptible to *Pst* DC3000, suggesting a positive role in disease resistance (Zhang et al., 2013). Increased expression of *CRK28* enhanced resistance to *Pst* DC3000 in Arabidopsis and induced cell death in *Nicotiana benthamiana* (Yadeta et al., 2017). In contrast, *CRK20* may regulate pathogenic defense in a negative way, as demonstrated by that its loss-of-function mutants increased resistance to *Pst* DC3000 (Ederli et al., 2011). Overexpression of *CRK4, CRK6*, and *CRK36* enhanced PTI responses in Arabidopsis, resulting in increased resistance to *Pst* DC3000 (Yeh et al., 2015). Several CRKs have been shown to be implicated in abiotic stress responses (Bourdais et al., 2015; Ramegowda and Senthil-Kumar, 2015). Knockdown of *CRK36* and knockout of *CRK45* increased sensitivity to abscisic acid (ABA) and osmotic stress in Arabidopsis (Tanaka et al., 2012). In addition, *crk5* mutant plants showed accelerated senescence and enhanced cell death phenotypes in response to UV radiation (Burdiak et al., 2015). It has been demonstrated that CRKs, including CRK5, CRK36, and CRK45, function in both biotic and abiotic stress responses.

In this study, *CRK36* was identified as one of highly expressed genes in response to the necrotrophic fungal pathogen *Alternaria brassicicola* in Arabidopsis. CRK36 overexpression in Arabidopsis enhanced hypersensitive cell death, ROS production, and disease resistance to *P. syringae*. In addition, flg22-triggered ROS burst and stomatal closure were increased in *CRK36*-overexpressing plants in a BIK1- and NADPH oxidase-dependent manner. CRK36 directly interacted with and induced phosphorylation of BIK1 in response to flg22 treatment. These results suggest that CRK36, in association with BIK1, plays an essential role in plant innate immunity by regulating ROS production and signaling of NADPH oxidases.

## Materials and methods

### Plant materials

*Arabidopsis thaliana* (ecotype Columbia, Col-0) plants were grown at 23°C under long-day conditions (16-h light/8-h dark cycle) for general growth and reproduction, and under short-day conditions (8-h light/16-h dark cycle) for pathogen infection. *N. benthamiana* plants were grown at 28°C under long-day conditions (16-h light/8-h dark cycle). The following mutant plants were used in this study: *crk36-1* (SALK_035659), *crk36-2* (SALK_100834), *crk36-3* (SALK_116300), *bik1* (Lu et al., 2010), and *rbohD/F* (Kwak et al., 2003). T-DNA insertion sites were verified by sequencing, and homozygous lines were selected. To generate *CRK36OE* plants, DNA fragments for *CRK36* were amplified from an Arabidopsis cDNA library by PCR and cloned into the pBI121 binary vector (Clonetech) under the control of the cauliflower mosaic virus (CaMV) 35S promoter. To generate *pCRK36:GUS* plants, the *CRK36* promoter region (−1 to −1,365 bp) was amplified from Arabidopsis gDNA by PCR and cloned into the pCAMBIA1303 vector containing a *GUS* gene. CRK36 mutants, *CRK36K*^*K268E*^, *CRK36*^*C12345A*^, and *CRK36*^*C6789A*^, were generated by site-directed mutagenesis using the primers in Table S1. The mutated sequences were confirmed by sequencing. All constructs were transformed into Arabidopsis plants using the floral dip method (Clough and Bent, 1998). Transformants were selected on 1/2 MS medium containing 30 μg/mL kanamycin and homozygous T3 or T4 seeds were used for experiments.

### Plant treatments

For pathogen infection, 6-week-old plants grown under short-day conditions in an 8-h light/16-h dark cycle were used. Treatments with *A. brassicicola, P. syringae*, and *E. carotovora* were performed as previously described (Oh et al., 2005). For *A. brassicicola* infection, leaves were inoculated onto leaves by applying 10 μL of water or spore suspension (10^6^ spores/mL). For *P. syringae* infection, leaves were infiltrated with 10 μL of MgCl_2_ (10 mM) or bacterial suspensions (10^6^ cfu/mL). For *E. carotovora* infection, leaves were infiltrated with 10 μL of NaCl (0.9%) or bacterial suspension (10^7^ cfu/mL). For flg22 treatment, leaves were infiltrated with water or 1 μM flg22 for 4 h (Kwon et al., 2013). For hormone treatments, 6-week-old plants grown under short-day conditions (8-h light/16-h dark cycle) were sprayed with water (mock), SA (1 mM), ethephon (ET; 1.5 mM), and methyl jasmonate (MeJA; 50 μM) dissolved in water. The treated plants were maintained at 100% humidity for the indicated times.

### Gene expression analysis

Gene expression was determined by quantitative real-time RT-PCR using KAPA™ SYBR FAST qPCR master mix with gene-specific primers (Table S1) on a LightCycler 480 system (Roche), according to the manufacturer's protocol. The expression levels of tested genes were standardized to the constitutive expression level of *ELF1A* and calculated using the 2^−ΔΔt^ method (Schmittgen and Livak, 2008). Experiments were repeated at least 3 times with biologically independent samples.

### Microarray analysis

Leaves of 6-week-old plants were inoculated with 10 μL water or suspension of *A. brassicicola* spores (5 × 10^5^ spores/mL) and incubated for 6 h as previously described (Oh et al., 2005). To analyze the transcript levels in plants treated with *A. brassicicola*, Affymetrix ATH1 microarray analysis was carried out. The expression levels were normalized by global scaling using GENPLEX software (Istech, Korea). Differentially expressed genes with at least two-fold changes are listed in Table S2.

### Histochemical staining

For GUS staining, *pCRK36*:*GUS* plants were stained in a solution containing the substrate 5-bromo-4-chloro-3-indolyl-β-D-glucuronide (X-Gluc) at 37°C as previously described (Jefferson et al., 1987). Stained tissues were cleared by several washings of 70% ethanol and examined under a light microscope. H_2_O_2_ was detected by 3,3′-diaminobenzidine (DAB) staining as previously described (Torres et al., 2002). Leaves were placed in 1% DAB solution (Sigma-Aldrich) overnight and destained with 70% ethanol. Images were captured using a microscope (Leica EZ4D), converted to greyscale, and then inverted to black-and-white images. The brightness of the inverted images was measured using ImageJ software (NIH) to quantify the intensity of brown color. Trypan blue staining was performed as previously described (Bowling et al., 1997). Leaves were immersed in lactic acid-phenol-trypan blue solution (2.5 mg/mL trypan blue, 25% lactic acid, 23% water-saturated phenol, 25% glycerol) and boiled for 1 min. Stained leaves were placed in a 60% chloral hydrate solution and finally equilibrated with 50% glycerol. Callose deposition was visualized by aniline blue staining as previously described (Clay et al., 2009). Leaves were fixed in acetic acid:ethanol (1:3) and then stained with 5 mg mL^−1^ aniline blue for 1 h. Stained leaves were observed under a confocal microscope (Zeiss Imager.A2).

### Apoplastic ROS assay

ROS was measured as previously described (Smith and Heese, 2014). Leaf discs (0.5 cm^2^) were incubated in 10 μL of distilled water overnight in a 96-well plate. Water was then replaced by a reaction solution supplemented with 100 nM of flg22. H_2_O_2_ levels were measured using the ROS-Glo™ H_2_O_2_ Assay kit (Promega) in a luminometer (Luminoskan, Thermo LabSystems).

### Ion leakage assay

Ion leakage experiments were performed as previously described (MacKey et al., 2003). Leaves were infiltrated with bacterial suspensions of *Pst* DC3000 (*AvrRpm1*) at 2 × 10^8^ cfu/mL. Leaf discs (0.5 cm^2^) were washed with 30 mL of distilled water for 30 min and then transferred into 10 mL distilled water. Ion conductivity was measured over time using a conductivity meter (Thermo Scientific).

### Stomatal aperture measurement

Stomatal aperture was measured as previously described (Li et al., 2014). Leaf peels were collected from the abaxial side of 6-week-old plants and floated in a buffer (10 mM MES, pH 6.15, 10 mM KCl, and 10 μM CaCl_2_) for 2 h under light condition to ensure that most stomata were open prior to treatment. Stomatal images were captured using an optical microscope (Zeiss Imager.A2) and stomatal length and width were measured using ImageJ software (NIH).

### Yeast two-hybrid analysis

A yeast two-hybrid assay was performed with the GAL4 system (Clontech), according to the manufacturer's instructions. DNA regions corresponding to the kinase domains of CRK36, FLS2, CERK1, and BAK1, and the full-length of BIK1 were PCR-amplified with gene-specific primers (Table S1) and cloned into the pCR2.1-TOPO vector (Invitrogen). The constructs were subsequently transferred into the pGBKT7 and pGADT7 destination vectors, encoding the GAL4 DNA-binding domain (BD) and activation domain (AD), respectively. The resulting fusion constructs were transformed into the AH109 yeast strain. The transformants were grown on SD/-Leu-Trp medium, and then transferred to SD/-Ade-His-Leu-Trp medium. Transactivation activity was further evaluated based on the activity of α-galactosidase on SD/-Ade-His-Leu-Trp medium containing 40 mg/L of X-α-Gal.

### BiFC assay

To generate the BiFC constructs, *BIK1* and *CRK36* were PCR-amplified using gene-specific primers (Table S1). The PCR products were cloned into the pSPYNE-35S or pSPYCE-35S vector for split YFP N-terminal or C-terminal fragment expression (Walter et al., 2004), fusing BIK1 to the N-terminal fragment of YFP (BIK1-YFP^N^) and CRK36 to the C-terminal fragment of YFP (CRK36-YFP^C^). *Agrobacterium tumefaciens* C58C1 harboring the BiFC constructs was coinfiltrated into *N. benthamiana* leaves. For expression in Arabidopsis protoplasts, PCR constructs for *bZIP63, CRK36, FLS2*, and *BIK1* were cloned into the pUC-SPYNE or pUC-SPYCE vector, generating the recombinant vectors pUC-SPYNE:bZIP63, pUC-SPYCE:bZIP63, pUC-SPYCE:CRK36, pUC-SPYNE:FLS2, and pUC-SPYNE:BIK1. The constructs were cotransfected into Arabidopsis mesophyll protoplasts by polyethylene glycol transfection (Yoo et al., 2007). The transfected leaves and protoplasts were visualized using a confocal microscope (LSM 510 META, Zeiss) with excitation at 488 nm and emission at 543 nm.

### GST pull-down assay

A pull-down assay was performed as previously described (Huh et al., 2010), with some modifications. GST or GST-fused BIK1 (10 μg) was incubated with His-tagged CRK36 (10 μg) in a binding buffer (20 mM Tris-HCl, pH 7.4, 200 mM NaCl, and 1 mM EDTA) for 2 h at 4°C. Glutathione sepharose 4B beads (GE healthcare) were added, incubated for 2 h, and washed 3 times with a washing buffer (20 mM Tris-HCl, pH 7.4, 200 mM NaCl, 1 mM EDTA, and 0.5% Triton X-100). Bound proteins were eluted by boiling in 2X Laemmli sample buffer (100 mM Tris-HCl, pH 6.8, 10% SDS, 30% glycerol, 5% β-mercaptoethanol, and 0.02% bromophenol blue), separated by SDS-PAGE, and visualized by immunoblotting using anti-GST and anti-His antibodies (Abcam).

### Co-immunoprecipitation assay

Co-immunoprecipitation experiments were performed as previously described (Huh et al., 2010) with some modifications. Proteins were extracted from *N. benthamiana* leaves using an extraction buffer [50 mM Tris-HCl, pH 8.0, 150 mM NaCl, 1% Triton X-100, 0.5 % (w/v) sodium deoxycholate, 1 mM PMSF, and 1x protease inhibitor cocktail (Roche)] with gentle shaking for 1 h at 4°C. Lysates were centrifuged at 12,000 rpm for 10 min at 4°C, and the supernatant was incubated with anti-Myc antibody (Abcam) overnight at 4°C. Protein A sepharose beads (GE healthcare) were added and washed 3 times with an extraction buffer. Bound proteins were eluted by boiling in 2X Laemmli sample buffer (100 mM Tris-HCl, pH 6.8, 10% SDS, 30% glycerol, 5% β-mercaptoethanol, and 0.02% bromophenol blue), separated by SDS-PAGE, and visualized by immunoblotting using anti-Myc and anti-HA antibodies (Abcam).

### BIK1 phosphorylation assay

BIK1 phosphorylation was determined by a BIK1 mobility shift as previously described (Lu et al., 2010). Protoplasts were transfected with BIK1-HA construct and treated with 1 μM flg22 for 15 min. Total proteins were extracted using an extraction buffer [10 mM HEPES, pH 7.5, 100 mM NaCl, 1 mM EDTA, 10% glycerol, 0.5% Triton X-100, and 1x protease inhibitor cocktail (Roche)], and treated with calf intestine phosphatase (New England Biolabs) to trigger dephosphorylation. Extracted proteins were subjected to boiling in 2X Laemmli sample buffer (100 mM Tris-HCl, pH 6.8, 10% SDS, 30% glycerol, 5% β-mercaptoethanol, and 0.02% bromophenol blue), separated by SDS-PAGE, and visualized by immunoblotting with anti-HA antibody (Abcam).

## Results

### Identification of CRK36 as an *A. brassicicola*-responsive gene

Kinases are essential molecules in signaling processes, and thus, we searched for kinases implicated in immune responses using a microarray analysis of transcriptional responses to *A. brassicicola* infection. *A. brassicicola* inoculation led to the identification of 224 genes with differential expression (≥2-fold change; *P* < 0.05; Table S2), which included 10 genes encoding distinct types of kinases (Table S3). Recent studies have demonstrated the implications of CRKs to immune responses (Wrzaczek et al., 2010; Bourdais et al., 2015; Yeh et al., 2015), and thus, we chose *CRK36* (At4g04490) for further study.

We initially examined *CRK36* expression in response to SA, methyl jasmonate (MeJA), and ethephon (ET), the ethylene releaser, using *PR1, PDF1.2*, and *GLIP1* as positive controls (Figure S1A; Kim et al., 2013). *CRK36* was highly expressed in leaves after hormone treatments. Tissue-specific expression was then assessed in transgenic Arabidopsis plants harboring the *CRK36* promoter (*pCRK36*) fused to a β*-glucuronidase* (*GUS*) reporter gene. GUS staining showed expression in the hypocotyls and roots of seedlings (5 DAG), older (14 DAG), and senescing (36 DAG) leaves, and hydathodes at all growth stages (Figure S1B). GUS activity was also detected in reproductive organs such as sepals and septum tips. We then tested the responses of *pCRK36:GUS* plants to pathogen infection (Figure S1C). GUS signals were greatly increased upon treatments with pathogens including *A. brassicicola*, virulent and avirulent strains of *P. syringae, Pst* DC3000 and *Pst* DC3000 (*AvrRpm1*), suggesting that *CRK36* is involved in pathogenic responses.

To study the function of *CRK36*, we obtained 3 different T-DNA insertion lines (SALK_035659; *crk36-1*, SALK_100834; *crk36-2*, and SALK_116300; *crk36-3*) that carry T-DNA insertions in the *CRK36* promoter region (Figure S2A). In the qRT-PCR analysis, *CRK36* mRNAs were still detectable in these mutant lines with 30–50% reduction of transcripts, indicating that they were knockdown mutants (Figure S2B). We also prepared transgenic plants (*CRK36OE*) overexpressing *CRK36* under the control of the CaMV 35S promoter in which *CRK36* transcript levels were significantly increased (Figure S2C).

### CRK36 regulates cell death, ROS production, and disease resistance in response to necrotrophic pathogens

**Figure 1:**
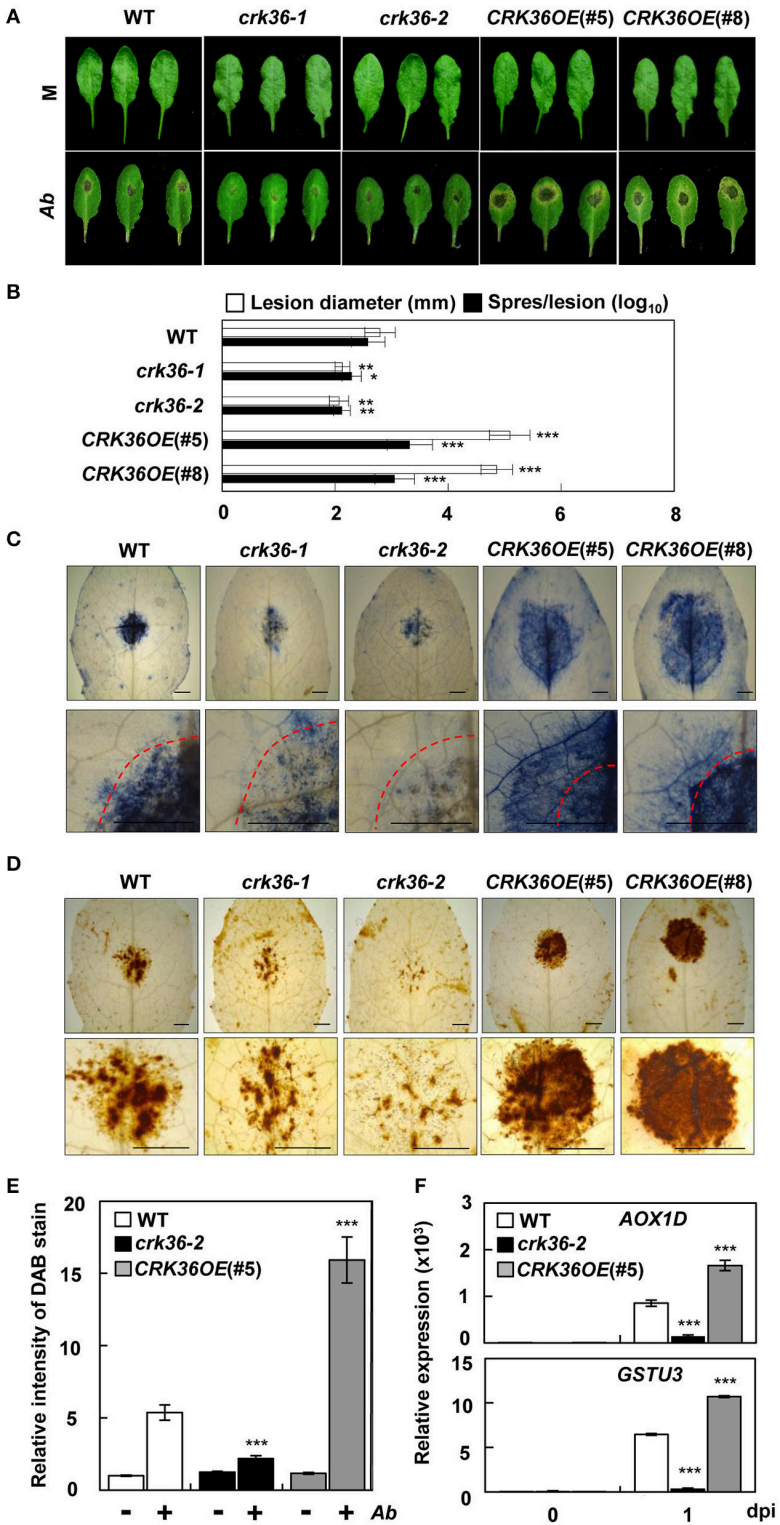
Responses of *crk36* and *CRK36OE* plants to the fungal pathogen *A. brassicicola*. **(A)** Disease symptoms in leaves inoculated with *A. brassicicola*. **(B)** Measurements of lesion diameter and spore number in leaves. Values are means ± SD (*n* = 6). **(C)** Necrotic lesions in leaves stained with trypan blue. Dashed red lines indicate the inoculation areas. **(D)** ROS production in leaves as determined by DAB staining. **(E)** Quantification of DAB-stained ROS in **(D)**. Values are means ± SD (*n* = 6). **(F)** qRT-PCR analysis of *AOX1D* and *GSTU3* expression. Results represent means (± SD) of 3 biological replicates. Leaves were inoculated with water (mock) or fungal spore suspension (1 × 10^6^ spores/mL) for 6 days in **(A–E)** or 1 day in **(F)**. Asterisks indicate significant differences from WT (*t*-test; ^*^P < 0.05; ^**^P < 0.01; ^***^P < 0.001). Experiments were repeated 3 times with similar results. M, mock. *Ab, A. brassicicola*. dpi, days post-inoculation. Bars, 5 mm.

We investigated how *crk36* and *CRK36OE* plants respond to pathogen infection. Since *CRK36* was identified as an *A. brassicicola*-responsive gene, plants were first challenged with *A. brassicicola* and assessed for disease development. In *CRK36OE* plants, necrotic cell death symptoms were enhanced, as indicated by increased lesion size (Figures 1A,B). The extent of cell death was further confirmed by trypan blue staining that marks dead cells (Figure 1C). In contrast, reduction of *A. brassicicola*-induced cell death and disease symptoms was observed in *crk36* knockdown lines. For quantitative evaluation of *A brassicicola* growth, the spore number and abundance of fungal DNA (internal transcribed spacers, ITS) were determined in the infected leaves. *CRK36OE* plants displayed susceptible phenotypes with increased spore number and ITS abundance (Figure 1B, Figure S3). We additionally tested susceptibility of *crk36-2* and *CRK36OE* plants to the compatible necrotrophic bacterial pathogen *Erwinia carotovora*. Compatible interaction developed in wild type plants, and it was enhanced in *CRK36OE* plants but suppressed in *crk36-2* plants (Figure S4).

We next carried out DAB staining to test whether cell death was correlated with ROS accumulation in the infected leaves. ROS production was greatly increased in *CRK36OE* plants but decreased in *crk36-2* plants following *A. brassicicola* inoculation (Figures 1D,E). As an initial step to explore the function of *CRK36*, we analyzed ATTED-II (http://atted.jp/), a gene coexpression database that provides information on gene interactions and coregulated gene networks (Figure S5; Obayashi et al., 2007). The list of coexpressed genes of *CRK36* included redox system-related genes such as *alternative oxidase 1D* (*AOX1D*) and *glutathione S-transferase TAU 3* (*GSTU3*) (Dixon et al., 2002; Konert et al., 2015). In the qRT-PCR analysis, *AOX1D* and *GSTU3* were both induced in wild type plants by *A. brassicicola* treatment (Figure 1F). In accordance with the positive correlation between CRK36 and ROS production, their expression further increased in *CRK36OE*, but decreased in c*rk36-2* plants. These results suggest that CRK36 may be implicated in cell death and redox regulation during immune responses.

### CRK36 regulates cell death, ROS production, and disease resistance in response to hemibiotrophic pathogens

**Figure 2:**
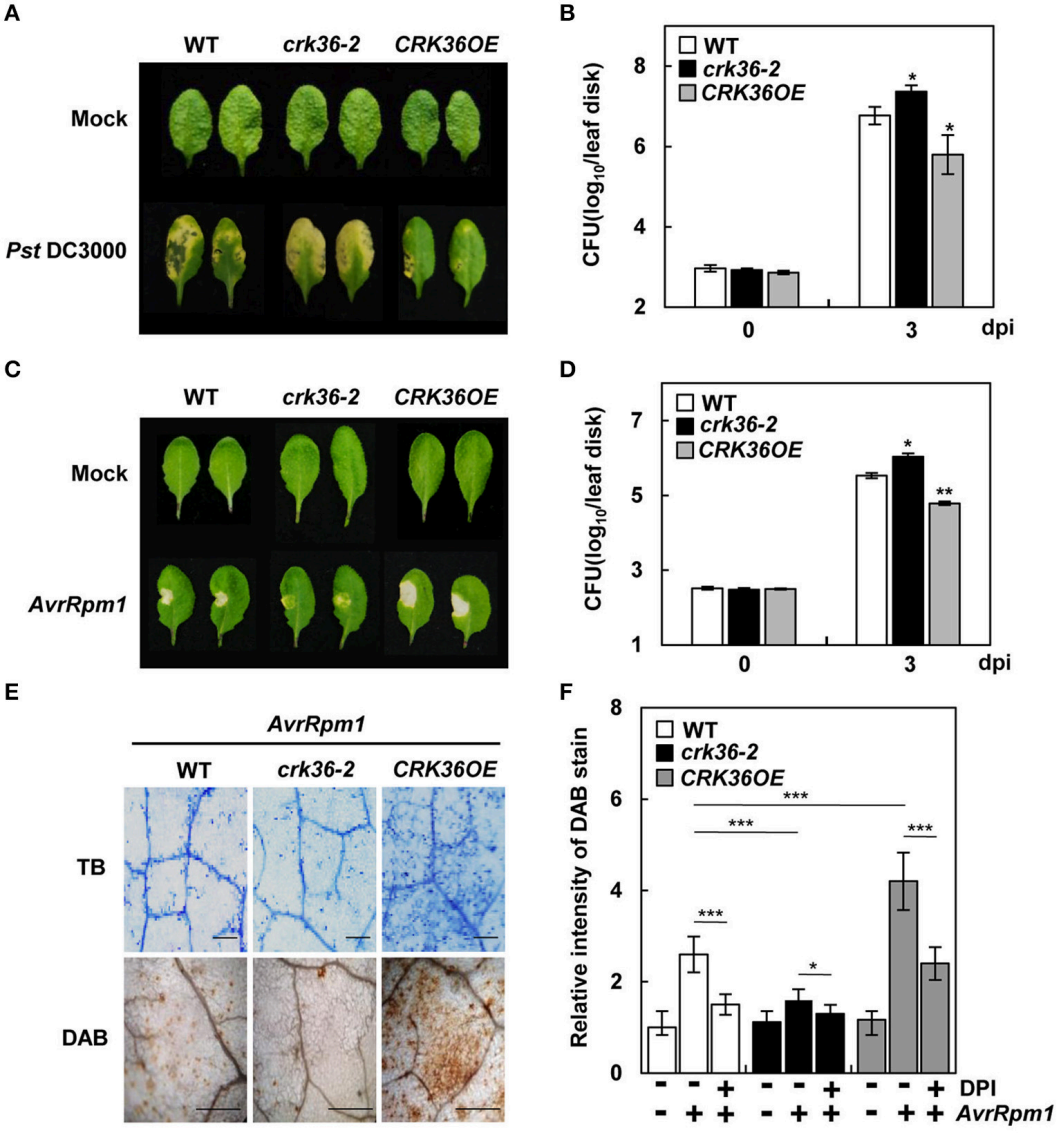
Responses of *crk36* and *CRK36OE* plants to the bacterial pathogens *Pst* DC3000 and *Pst* DC3000 (*AvrRpm1*). **(A,C)** Disease symptoms in leaves inoculated with *Pst* DC3000 **(A)** and *Pst* DC3000 (*AvrRpm1*) **(C)**. **(B,D)** Bacterial growth in leaves inoculated with *Pst* DC3000 **(B)** and *Pst* DC3000 (*AvrRpm1*) **(D)**. Values are means ± SD (*n* = 9). **(E)** Cell death determined by trypan blue (TB) staining (top) and ROS production by DAB staining (bottom) after *Pst* DC3000 (*AvrRpm1*) treatment. **(F)** Quantification of DAB-stained ROS in **(E)** and DPI effect on ROS production after *Pst* DC3000 (*AvrRpm1*) treatment. Leaves were pre-treated with 10 μM DPI for 1 h and then infected with pathogens. Values are means ± SD (*n* = 6). Leaves were inoculated with *Pst* DC3000 or *Pst* DC3000 (*AvrRpm1*) at 1 × 10^6^ cfu/mL for 3 days in **(A–D)** and with *Pst* DC3000 (*AvrRpm1*) at 1 × 10^7^ cfu/mL for 1 day in **(E,F)**. Asterisks indicate significant differences from WT in **(B,D,F)** and between untreated and DPI-treated plants in **(F)** (*t*-test; ^*^P < 0.05; ^**^P < 0.01; ^***^P < 0.001). Experiments were repeated 3 times with similar results. dpi, days post-inoculation.

Among the tested independent lines, *crk36*-2 and *CRK36OE*(#5) were chosen for further analysis. We next challenged plants with virulent and avirulent bacterial pathogens *Pst* DC3000 and *Pst* DC3000 (*AvrRpm1*). In contrast to the resistance response to *A. brassicicola*, disease symptoms were more severe in *crk36-2* plants but were reduced in *CRK36OE* plants upon *Pst* DC3000 infiltration, which correlated with bacterial growth (Figures 2A,B). Similar bacterial growth results were obtained with *Pst* DC3000 (*AvrRpm1*) (Figure 2D). As observed upon *A. brassicicola* infection, HR PCD developed more strongly in *Pst* DC3000 (*AvrRpm1*)-infiltrated *CRK36OE* leaves but more weakly in *crk36-2* than in wild type (Figure 2C). Cell death phenotypes of plants were confirmed by trypan blue staining and electrolyte leakage assay (Figure 2E, Figure S6). Consistently, significant induction of pathogenesis-related (PR) genes *PR1, PR2*, and *PR5* was observed in *CRK36OE* plants in response to both *Pst* DC3000 and *Pst* DC3000 (*AvrRpm1*); this was reversed in *crk36-2* plants (Figure S7). Another avirulent bacterial pathogen *Pst* DC3000 (*AvrRpt2*) was also tested. *Pst* DC3000 (*AvrRpt2*) infiltration evoked HR cell death and trypan blue staining patterns in the infected *crk36-2* and *CRK36OE* leaves similarly to *Pst* DC3000 (*AvrRpm1*) (Figure S8A).

ROS production is an early response of HR, and has been attributed to NADPH oxidases (Tang et al., 1998; Torres et al., 2002, 2006). Thus, we monitored ROS production in plants challenged with either *Pst* DC3000 (*AvrRpm1*) or *Pst* DC3000 (*AvrRpt2*) by DAB staining. *CRK36OE* plants accumulated more DAB-stained ROS than wild type, whereas *crk36-2* plants showed lower ROS production (Figures 2E,F, Figures S8A,B). Treatment with diphenylene iodonium (DPI), an NADPH oxidase inhibitor, reduced avirulent pathogen-induced ROS accumulation in all plants (Figure 2F, Figure S8B), which agreed with previous reports that NADPH oxidases are the primary ROS-generating system during incompatible plant-pathogen interactions. These results suggest that CRK36 positively regulates HR cell death, its associated ROS production, and ultimately, ETI responses.

### Function of CRK36 in cell death processes

We investigated whether CRK36 is also implicated in other PCD processes. Plants were treated with the PCD-eliciting fungal toxin fumonisin B1 (FB1) (Kwon et al., 2009). As observed with pathogen infection, the extent of FB1-triggered cell death was increased in FB1-treated *CRK36OE* leaves, in contrast to *crk36-2* leaves, which showed reduced cell death lesions (Figure S9A). In addition, we determined the involvement of *CRK36* in leaf senescence, a developmental PCD process (Quirino et al., 2000; Lim et al., 2007). During dark-induced senescence, *GUS* expression driven by *pCRK36* gradually increased, and yellowing was accelerated in *CRK36OE* leaves but delayed in *crk36-2* leaves (Figures S9B,C). Age-dependent leaf senescence was also monitored. *CRK36OE* plants exhibited an early-senescence phenotype, which was delayed in *crk36-2* leaves (Figure S9D). These results suggest that CRK36 is involved in various types of PCD processes in plants.

### CRK36 regulates PTI responses

**Figure 3:**
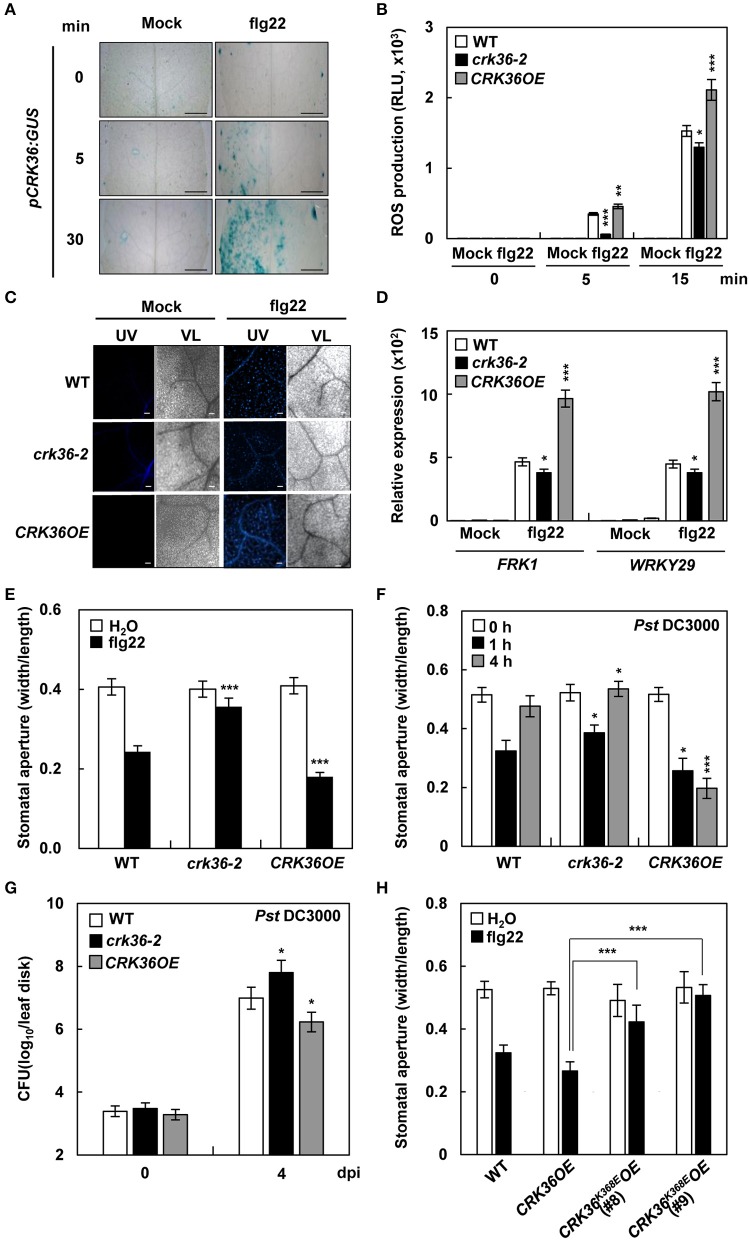
PTI responses in *crk36* and *CRK36OE* plants. **(A)** GUS activity in *pCRK36:GUS* leaves in response to flg22 (1 μM). Bars, 1 mm. **(B)** flg22-triggered oxidative burst. Leaf discs were taken from water- or flg22 (100 nM)-treated plants and monitored for ROS generation (presented as total photon count). Values are means ± SD (*n* = 10). **(C)** Callose deposition. Leaves were treated with water (mock) or flg22 (1 μM) for 18 h and stained with aniline blue. UV, ultraviolet light. VL, visible light. Bars, 100 μm. **(D)** qRT-PCR analysis of PTI marker genes *FRK1* and *WRKY29*. Results represent means (±SD) of 3 biological replicates. **(E)** flg22-induced stomatal movement. Stomatal apertures were measured in epidermal peels floated in MES buffer containing flg22 (1 μM) for 2 h. Values are means ± SD (*n* = 60). **(F)** Pathogen-induced stomatal movement. Stomatal apertures were measured in epidermal peels of plants after spray-inoculation with *Pst* DC3000 (1 × 10^8^ cfu/mL). Values are means ± SD (*n* = 60). **(G)** Bacterial growth in leaves spray-inoculated with *Pst* DC3000 (5x10^8^ cfu/mL). Values are means ± SD (*n* = 3). **(H)** flg22-induced stomatal movement in kinase-dead *CRK36* transgenic plants. Stomatal apertures were measured in epidermal peels floated in MES buffer containing flg22 (1 μM) for 2 h. Values are means ± SD (*n* = 60). Asterisks indicate significant differences from WT in **(C-G)** and from *CRK36OE* in **(H)** (*t*-test; ^*^P < 0.05; ^**^P < 0.01; ^***^P < 0.001). Experiments were repeated 3 times with similar results.

It was then investigated whether CRK36 plays a role in PTI responses, including ROS production, callose deposition, and gene expression. We first examined *CRK36* activation in response to a typical PAMP flg22. Transcriptional response of *CRK36* to flg22 was rapid, as GUS signals were detected in *pCRK36:GUS* plants 5 min after flg22 treatment (Figure 3A). ROS production, callose deposition, and expression of PTI responsive genes *FRK1* and *WRKY29* were markedly increased in flg22-treated *CRK36OE* plants but somewhat decreased in *crk36-2* plants as compared to that in wild type plants (Figures 3B–D). Stomatal closure is an essential PTI response, known as stomatal immunity, that is triggered by a PAMP-triggered ROS burst (Melotto et al., 2006). In line with flg22-triggered ROS production, flg22 treatment induced stomatal closure in wild type plants, and this was impaired in *crk36-2* mutant (Figure 3E). However, in *CRK36OE* plants, stomata were more strongly closed after flg22 treatment. While stomatal closure is an important early event to prevent bacterial entry, *Pst* DC3000 promotes the re-opening of stomata and thus the invasion of leaf tissues (Zeng and He, 2010). We measured stomatal apertures following surface-inoculation with *Pst* DC3000. Stomatal closure of plants incubated with pathogens for 1 h was similar to that induced by flg22 (Figure 3F). Stomatal re-opening was observed 4 h after exposure to *Pst* DC3000, and was more pronounced in *crk36-2* mutant but completely abolished in *CRK36OE* plants. Following surface inoculation with *Pst* DC3000, bacterial populations were higher in *CRK36OE* leaves but lower in *crk36-2* mutant than in wild type plants (Figure 3G). We additionally prepared transgenic plants (*CRK36*^K386E^*OE*) overexpressing a kinase-dead CRK36^K386E^ mutant form in which the conserved Lys386 was replaced by Asp (Figure S10A). Overexpression of *CRK36*^K386E^ attenuated flg22-induced stomatal closure (Figure 3H), suggesting that CRK36^K386E^ exerts a dominant negative effect and that CRK36 requires kinase activity for its function in basal immune responses. Together, these results demonstrate that CRK36 positively regulates PTI, including stomatal defense.

### Conserved Cys residues in the DUF26 domains are essential for CRK36 functions

**Figure 4:**
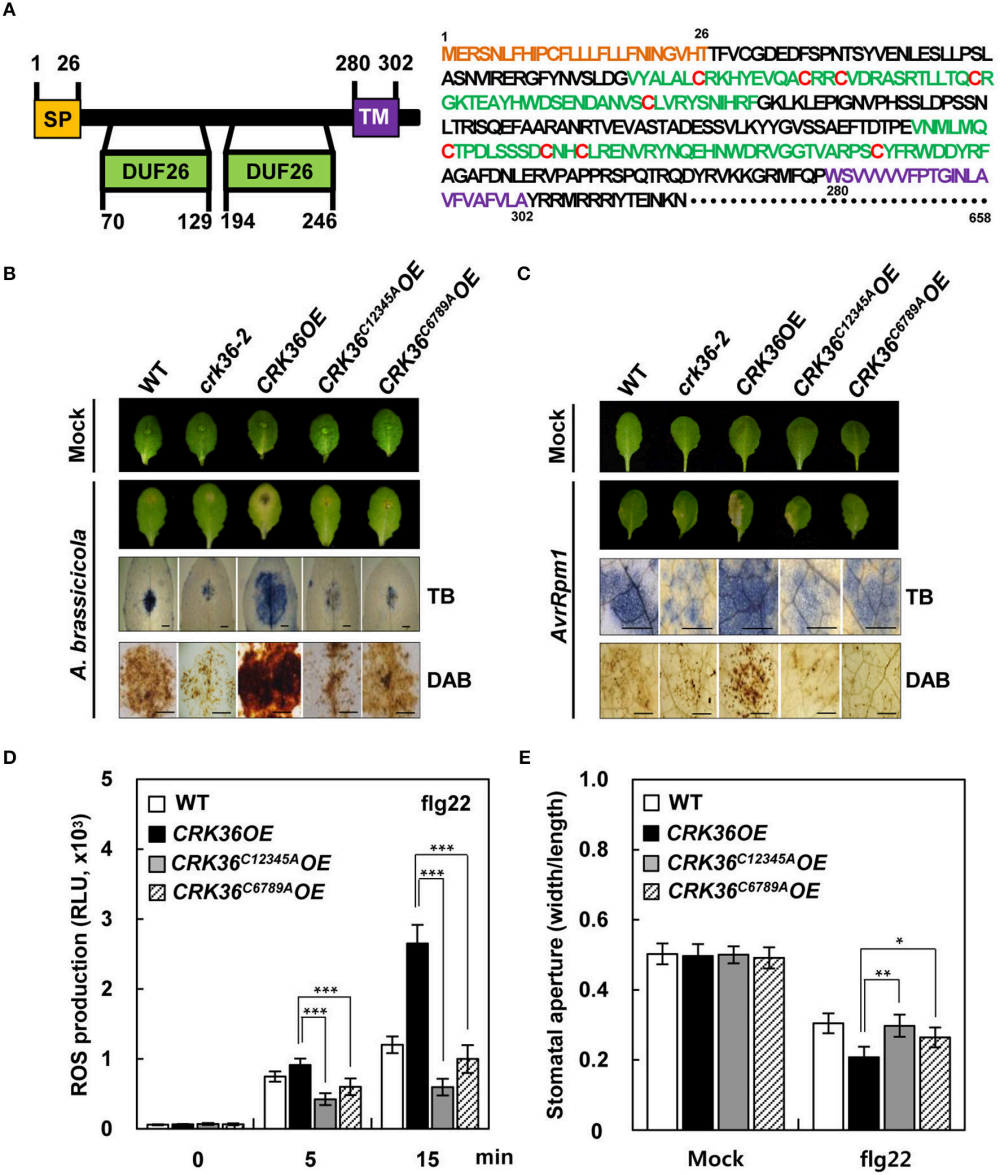
Effect of Cys mutations in DUF26 motifs of CRK36 on immune responses. **(A)** Schematic structure (left) and amino acid sequence (right) of CRK36 protein. Signal peptide (SP), DUF26 motifs, transmembrane domain (TM), and conserved Cys residues are indicated in yellow, green, purple, and red colors, respectively. The diagram of the CRK36 structure was made using the ExPASY Proteomics Server PROSITE module. **(B,C)** Cell death and ROS production in *CRK36*^*C12345A*^*OE* and *CRK36*^*C6789A*^*OE* plants challenged with pathogens. Phenotypes of leaves, cell death determined by trypan blue (TB) staining, and ROS production by DAB staining after *A. brassicicola* **(B)** and *Pst* DC3000 (*AvrRpm1*) **(C)** treatments. **(D)** flg22-triggered oxidative burst. Leaf discs were taken from flg22 (100 nM)-treated plants and monitored for ROS generation (presented as total photon count). Values are means ± SD (*n* = 9). **(E)** flg22-induced stomatal movement. Stomatal apertures were measured in epidermal peels floated in MES buffer containing flg22 (1 μM) for 2 h. Values are means ± SD (*n* = 60). Asterisks indicate significant differences from *CRK36OE* (*t*-test; ^*^P < 0.05; ^**^P < 0.01; ^***^P < 0.001). Experiments were repeated 3 times with similar results.

CRK36 contains 9 conserved Cys residues in 2 DUF26 motifs of the extracellular region: 5 in the first DUF26 motif and the other 4 in the second DUF26 motif (Figure 4A). As the thiol side chain in Cys is susceptible to oxidation, CRKs have been suggested as potential targets for redox regulation (Wrzaczek et al., 2010; Bourdais et al., 2015). To explore the role of these conserved Cys residues, we made 2 mutant constructs, CRK36^C12345A^ and CRK36^C6789*A*^, in which 5 and 4 Cys residues of the first and second DUF26 motifs, respectively, were changed to Ala residues. We then prepared transgenic Col-0 plants overexpressing them (*CRK36*^*C12345A*^*OE* and *CRK36*^*C6789A*^*OE*) (Figure S10B). Among these, *CRK36*^*C12345A*^*OE*(#6) and *CRK36*^*C6789A*^*OE*(#1) were used for further analysis. When inoculated with either *A. brassicicola* or *Pst* DC3000 (*AvrRpm1*), the enhancement of hypersensitive cell death lesions and ROS production in *CRK36OE* plants was not observed in *CRK36*^*C12345A*^*OE* and *CRK36*^*C6789A*^*OE* leaves, which exhibited hypersensitive responses similar to those in wild type (Figures 4B,C). We also tested whether Cys mutations affected PTI responses. Likewise, positive effects of *CRK36* overexpression on flg22-induced ROS burst and stomatal closure were abolished in *CRK36*^*C12345A*^*OE* and *CRK36*^*C6789A*^*OE* plants (Figures 4D,E). These results suggest that Cys residues in the DUF26 domains are essential for CRK36 functions in PTI and ETI, particularly in ROS production events.

### CRK36 physically interacts with BIK1

**Figure 5:**
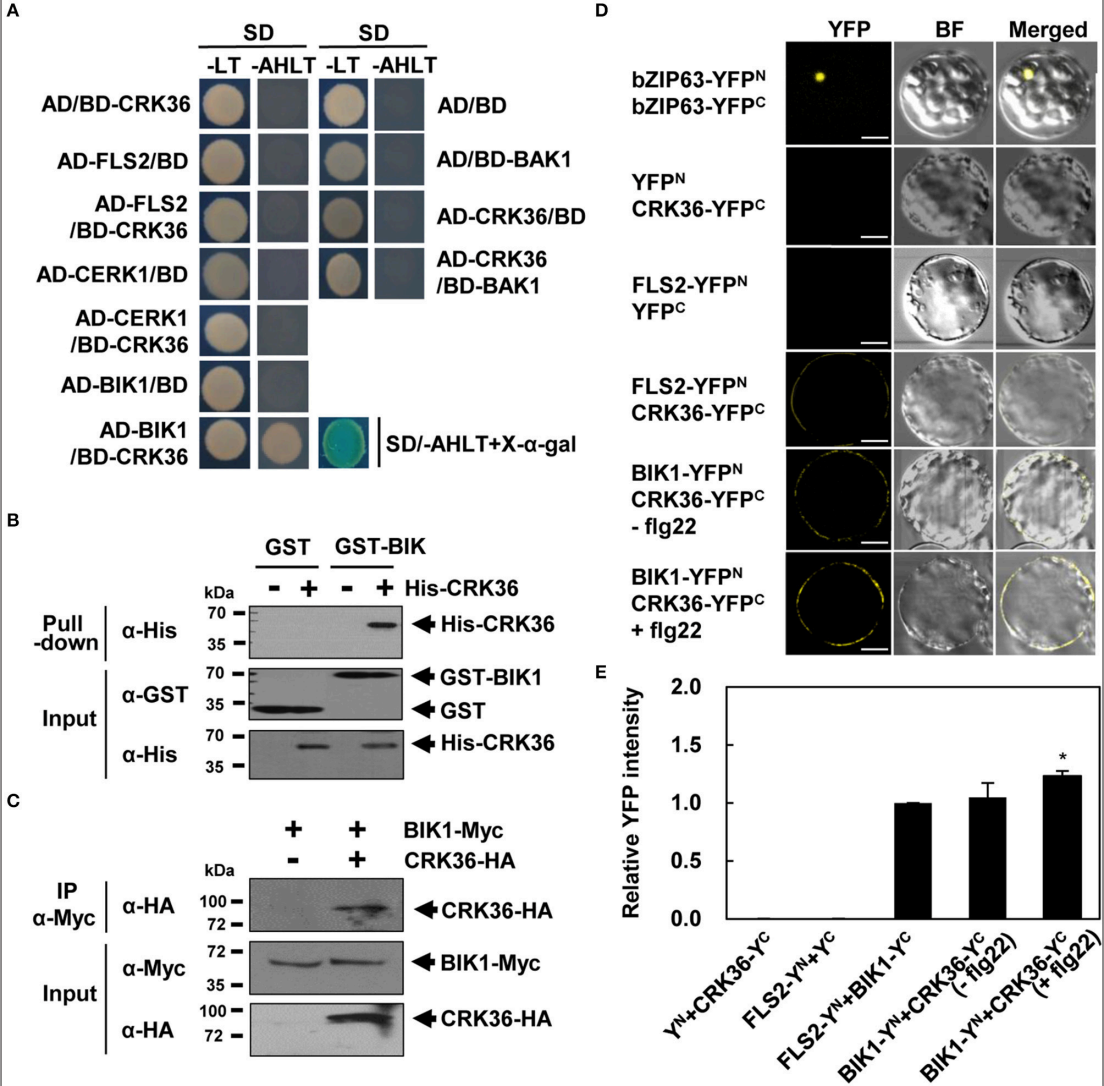
Physical interaction between CRK36 and BIK1. **(A)** Yeast two-hybrid assay for interaction of CRK36 with PTI components. Cytoplasmic kinase domains of FLS2, CERK1, BAK1, and CRK36 and the full-length of BIK1 were fused with GAL4 BD or AD as indicated. Their interactions were tested on selective medium lacking adenine, histidine, leucine and tryptophan (–AHLT) in the absence or presence of X-α-Gal. **(B)** *In vitro* GST pull-down assay for interaction between CRK36 and BIK1. GST or GST-fused BIK1 was incubated with the His-tagged kinase domain of CRK36 and precipitated with glutathione sepharose 4B beads. Proteins were detected by immunoblotting with anti-GST and anti-His antibodies. Input shows 1% of the amount used in binding reactions. **(C)** *In vivo* co-immunoprecipitation assay for interaction between CRK36 and BIK1. Protein extracts were prepared from *N. benthamiana* leaves expressing full-lengths of CRK36-HA and BIK1-Myc, and subjected to immunoprecipitation with anti-Myc antibody. Proteins were detected by immunoblotting with anti-HA and anti-Myc antibodies. Input shows 1% of the amount used in the binding reactions. IP, immunoprecipitation. **(D)** BiFC assay for interaction of CRK36 with BIK1 and FLS2 in Arabidopsis protoplasts. YFP^N^, YFP^C^, and their fusions with bZIP63, CRK36, FLS2, and BIK1 were co-expressed in Arabidopsis protoplasts for 24 h, and then treated with 100 nM flg22 for 15 min as indicated. Reconstituted YFP fluorescence was visualized under a confocal microscope. BF, bright field. Bars, 10 μm. **(E)** Quantitative analysis of interaction of CRK36 with BIK1 and FLS2 in **(D)**. YFP fluorescence was quantified based on pixel intensity. Values are means ± SD (*n* = 12). Asterisk indicates significant difference between untreated and flg22-treated BIK1-YFP^n^+CRK36-YFP^c^ (*t*-test; ^*^P < 0.05). Y^N^,YFP^N^; Y^C^, YFP^C^. Experiments were repeated 3 times with similar results.

Since CRK36 plays a positive role in flg22-induced PTI, we investigated whether CRK36 interacts with the components of the flg22-sensing PRR complex, including FLS2, BAK1, and BIK1, in yeast two-hybrid assay. Chitin elicitor receptor kinase 1 (CERK1), a different PRR, was also included in the analysis. In the yeast two-hybrid system, the interacting proteins are located in the nucleus, and membrane proteins may form aggregates without membranes being provided. For this reason, we used the intracellular kinase domains of CRK36 and PRRs instead of the entire coding region. CRK36 and tested proteins fused to the GAL4-activation domain (AD) and DNA-binding domain (BD) were expressed pairwise in yeast cells (Figure 5A). Of these, yeast cells co-transformed with AD-BIK1 and BD-CRK36 constructs were able to grow on selective medium lacking adenine, histidine, leucine, and tryptophan, and turned blue in the presence of X-α-Gal, implying a direct interaction between CRK36 and BIK1. No interactions were observed between CRK36 and the other tested proteins, FLS2, CERK1, and BAK1. Expression of proteins was validated by western blotting of yeast cell extracts using anti-HA and anti-Myc antibodies (Figure S11). GST pull-down assay confirmed the *in vitro* interaction between CRK36 and BIK1 (Figure 5B).

To further evaluate the interaction *in vivo*, we carried out bimolecular fluorescence complementation (BiFC) experiments using full-length proteins. The constructs of BIK1 fused with the N-terminal part of YFP (BIK1-YFP^N^) and CRK36 fused with the C-terminal part of YFP (CRK36-YFP^C^) were prepared, and singly or in combination transiently expressed in *N. benthamiana* leaves (Figure S12). YFP fluorescence was only detected when BIK1-YFP^N^ and CRK36-YFP^C^ were co-expressed. Their interaction was further observed by the co-immunoprecipitation of BIK1 and CRK36 in tobacco leaf extracts (Figure 5C). While we were conducting this study, a report was released demonstrating that CRK36 associates with FLS2 in Arabidopsis protoplasts as shown by BiFC assay (Yeh et al., 2015). Therefore, BiFC assays were additionally performed in Arabidopsis protoplasts to assess the interactions of CRK36 with FLS2 and BIK1. In agreement with the former result, an YFP signal was observed in protoplasts co-transformed with FLS2-YFP^N^ and CRK36-YFP^C^ (Figure 5D). Co-expression of BIK1-YFP^N^ and CRK36-YFP^C^ also produced YFP fluorescence in the plasma membrane, and this signal was somewhat enhanced by flg22 treatment (Figure 5E). These results suggest that the intracellular kinase domains are not sufficient for the interaction between CRK36 and FLS2 and that CRK36, FLS2, and BIK1 are associated together in plants.

### CRK36 functionally interacts with BIK1 for stomatal defense

**Figure 6:**
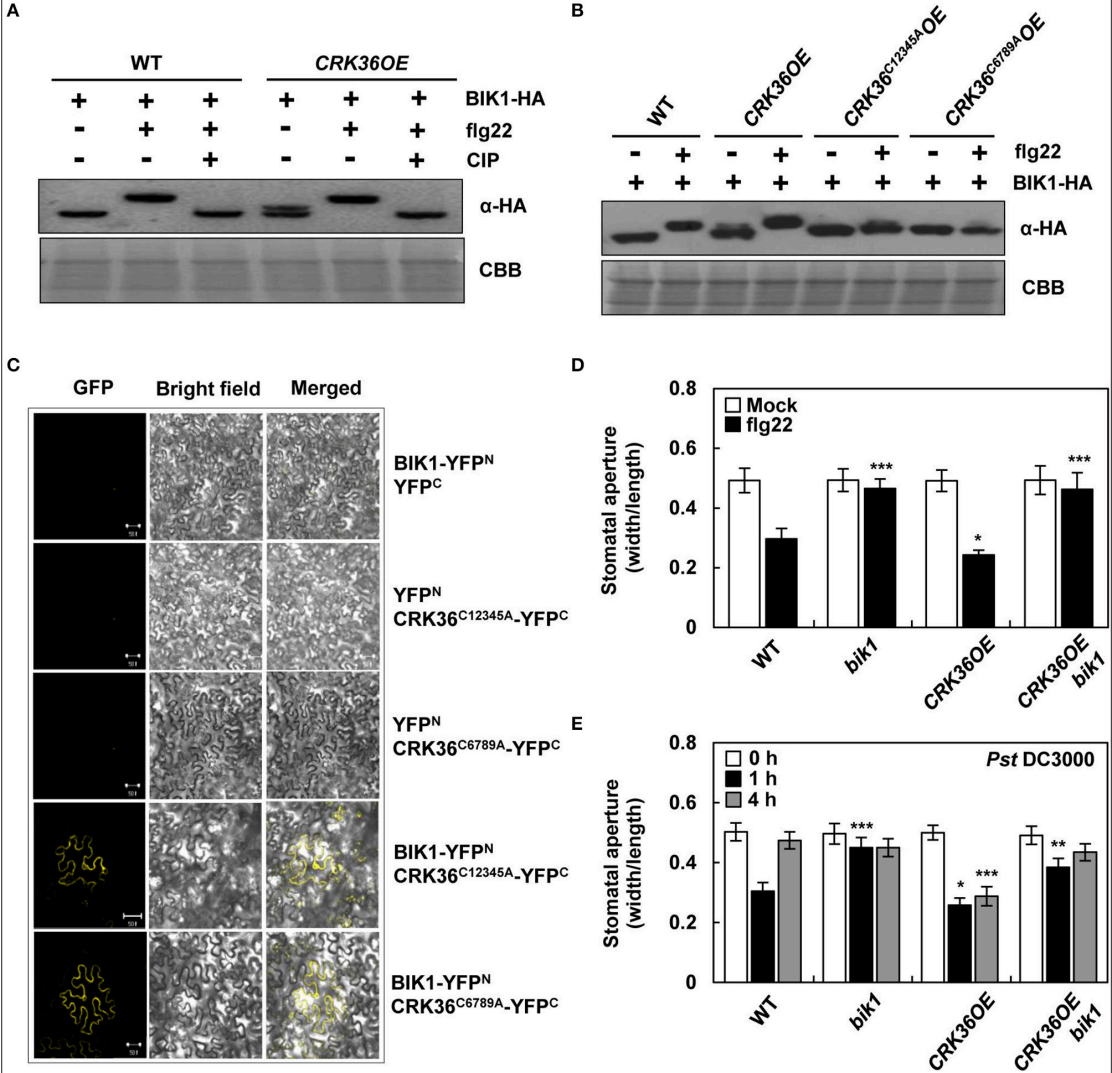
Functional interaction between CRK36 and BIK1. **(A)** flg22- and CRK36-induced BIK1 phosphorylation. BIK1-HA was expressed in wild type Col-0 and *CRK36OE* protoplasts and treated with 1 μM flg22 for 15 min. Protein extracts were treated with calf alkaline intestinal phosphatase (CIP) for BIK1 dephosphorylation and subjected to immunoblotting with anti-HA antibody for detection of BIK1 proteins. Comassie blue staining (CBB) served as a loading control. **(B)** Effect of Cys mutations in DUF26 motifs of CRK36 on BIK1 phosphorylation. BIK1-HA was expressed in wild type Col-0, *CRK36OE, CRK36*^*C12345A*^*OE*, and *CRK36*^*C6789A*^*OE* protoplasts and treated with 1 μM flg22 for 15 min. Protein extracts were subjected to immunoblotting with anti-HA antibody. CBB served as a loading control. **(C)** Effect of Cys mutations in DUF26 motifs of CRK36 on interaction with BIK1. For BiFC assay, YFP^N^, YFP^C^, BIK1-YFP^N^, CRK36^C12345A^-YFP^C^, and CRK36^C6789A^-YFP^C^ were co-expressed in *N. benthamiana* leaves for 24 h as indicated. Reconstituted YFP fluorescence was visualized under a confocal microscope. Bars, 50 μm. **(D)** Effect of *BIK1* mutation on flg22- and CRK36-induced stomatal closure. Stomatal apertures were measured in epidermal peels floated in MES buffer containing flg22 (1 μM) for 2 h. Values are means ± SD (*n* = 60). **(E)** Effect of *BIK1* mutation on pathogen- and CRK36-induced stomatal closure. Stomatal apertures were measured in epidermal peels of plants after spray-inoculation with *Pst* DC3000 (1 × 10^8^ cfu/mL). Values are means ± SD (*n* = 60). Asterisks indicate significant differences from WT (*t*-test; ^*^P < 0.05; ^**^P < 0.01; ^***^P < 0.001). Experiments were repeated 3 times with similar results.

It has been previously demonstrated that FLS2-BAK1-associated BIK1 is phosphorylated upon flagellin perception and in turn phosphorylates the NADPH oxidase, enhancing ROS generation (Lu et al., 2010; Kadota et al., 2014; Li et al., 2014). Accordingly, the interaction between CRK36, FLS2, and BIK1 led us to hypothesize that the CRK36-mediated ROS burst during PTI may result from stepwise reactions, i.e., CRK36-mediated BIK1 activation, BIK1-mediated phosphorylation of NADPH oxidases, and NADPH oxidase-mediated ROS production. To test our hypothesis, we first examined whether CRK36 triggers BIK1 phosphorylation. PAMP-induced BIK1 phosphorylation has been shown by a mobility shift in western blotting (Lu et al., 2010). Protoplasts derived from wild type and *CRK36OE* plants were prepared and transformed with the hemagglutinin (HA)-tagged BIK1 construct. BIK1 protein expression was visualized by immunoblotting using anti-HA antibody (Figure 6A). *CRK36OE* protoplasts constitutively expressed marginally slower-migrating BIK1 protein, and the mobility shift was prominent upon flg22 treatment in both wild type and *CRK36OE* protoplasts. Treatment with calf alkaline intestinal phosphatase (CIP) restored the mobility shift of BIK1, suggesting that CRK36 triggers BIK1 phosphorylation in PTI responses.

Given that conserved Cys residues in the DUF26 domains are essential for CRK36 functions in PTI (Figure 4), we checked the mobility of BIK1 bands in *CRK36*^*C12345A*^*OE* and *CRK36*^*C6789A*^*OE* protoplasts. While similar slightly slower BIK1 was observed, flg22-induced large shift in flg22-treated wild type and *CRK36OE* protoplasts was abolished in *CRK36*^*C12345A*^*OE* and *CRK36*^*C6789A*^*OE* protoplasts (Figure 6B). Multiple phosphorylation sites have been identified in BIK1 after BIK1 autophosphorylation and BAK1 transphosphorylation reactions (Lin et al., 2014), implying that overexpression of CRK36, either in wild type or in Cys mutant forms, triggered part of BIK1 phosphorylation events. BiFC experiments were further performed by co-expressing BIK1-YFP^N^ and either CRK36^C12345A^-YFP^C^ or CRK36^C6789A^-YFP^C^ in tobacco leaves. YFP fluorescence was produced in the plasma membrane, demonstrating that Cys mutant forms of CRK36 retain the ability to associate with BIK1 (Figure 6C). These results suggest that the inability of *CRK36*^*C12345A*^*OE* and *CRK36*^*C6789A*^*OE* plants to trigger PTI responses is due to the defect in BIK1 phosphorylation and that CRK36-triggered BIK1 phosphorylation is important for the role of CRK36 in basal immunity.

**Figure 7:**
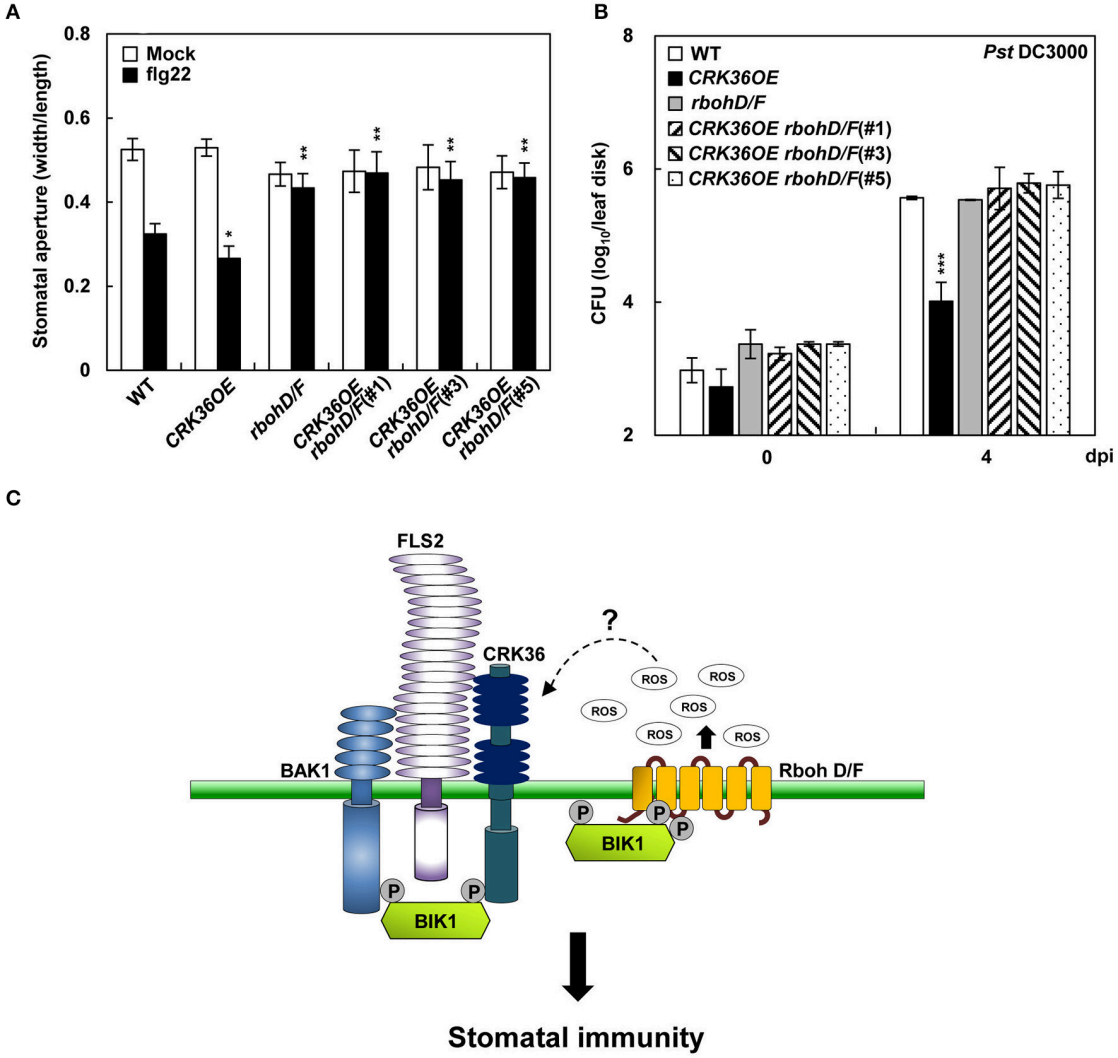
Stomatal immune responses in *CRK36OE, rbohD/F*, and *CRK36OE rbohD/F* plants. **(A)** Effect of *RbohD/F* mutations on flg22- and CRK36-induced stomatal closure. Stomatal apertures were measured in epidermal peels floated in MES buffer containing flg22 (1 μM) for 2 h. Values are means ± SD (*n* = 60). Asterisks indicate significant differences from WT (*t*-test; ^*^P < 0.05; ^**^P < 0.01). **(B)** Effect of *RbohD/F* mutations on CRK36-induced pathogen resistance. Bacterial growth was determined in leaves spray-inoculated with *Pst* DC3000 (5 × 10^8^ cfu/mL). Values are means ± SD (*n* = 6). Asterisks indicate significant differences from WT (*t*-test; ^***^P < 0.001). **(C)** A model for CRK36 function in stomatal immunity. BIK1 is phosphorylated and activated when flg22 binds to the FLS2-BAK1 complex. The activated BIK1 in turn phosphorylates NADPH oxidase, triggering ROS generation. We propose that CRK36 acts as an ROS receptor that is activated by sensing ROS. The activated CRK36 enhances BIK1 phosphorylation, boosting NADPH oxidase-mediated ROS burst. CRK36, BIK1, NADPH oxidase, and ROS may form a feedback activation loop regulating stomatal defense against pathogens.

To examine whether CRK36 functionally depends on BIK1, we crossed *CRK36OE* with *bik1* mutant and determined stomatal defense. The flg22- and *Pst* DC3000-triggered stomatal closure and prevention of stomatal re-opening following *Pst* DC3000 infection observed in *CRK36OE* plants were impaired in *CRK36OE bik1* plants (Figures 6D,E). As BIK1 regulates stomatal immunity through direct phosphorylation of the NADPH oxidase RbohD (Kadota et al., 2014; Li et al., 2014), we further crossed *CRK36OE* and *rbohD/F* (*rbohD rbohF* double mutant) and examined stomatal defense phenotypes. As expected, *CRK36OE rbohD/F* plants lost both flg22-induced stomatal closing and resistance to *Pst* DC3000 (Figures 7A,B). These results demonstrate that BIK1 and NADPH oxidases are required for CRK36 function in stomatal immunity.

## Discussion

Using cDNA microarray analysis, we identified *CRK36* as an *A. brassicicola*-responsive gene. *CRK36OE* plants showed enhanced cell death and ROS production in response to avirulent pathogens including *A. brassicicola*. Disease resistance to *Pst* DC3000 was increased in *CRK36OE* plants and this was associated with NADPH oxidase-mediated ROS burst and stomatal closure. CRK36 directly interacted with and required BIK1 for stomatal defense, and further triggered BIK1 phosphorylation. Our results suggest that CRK36 plays an essential role in innate immunity through the regulation of ROS production in a BIK1- and NADPH oxidase-dependent manner.

### Disease resistance and cell death

CRKs form one of the largest RLK subfamilies, and play important roles in the regulation of defense responses and PCD in Arabidopsis (Chen et al., 2003; Acharya et al., 2007; Ederli et al., 2011). In previous reports, Arabidopsis plants overexpressing *CRK* genes enhanced resistance to *Pst* DC3000 and activated rapid HR cell death, suggesting that CRKs function as positive regulators in disease resistance (Acharya et al., 2007; Zhang et al., 2013; Yeh et al., 2015). While the involvement of CRKs in resistance to necrotrophic fungal pathogens has not previously been studied, microarray analysis showed that the expression of a number of *CRKs*, including *CRK36*, was altered by treatments with fungal pathogens, the biotrophic powdery mildew *Golovinomyces orontii* and the non-host powdery mildew *Blumeria graminis* f. sp. *hordei* (Bourdais et al., 2015).

In our study, *CRK36* was transcriptionally regulated by *A. brassicicola*, and *CRK36OE* plants were susceptible to *A. brassicicola* and *E. carotovora*, in contrast to wild type and *crk36* plants. Conversely, *CRK36* overexpression increased resistance to *Pst* DC3000 and *Pst* DC3000 (*AvrRpm1*), decreasing bacterial growth in inoculated leaves. Our results suggest that disease resistance phenotypes may be correlated with a positive role of CRK36 in cell death. Pathogen-induced PCD has been suggested as a mechanism to restrict the growth and spread of pathogens to other uninfected tissues, as observed in *Pst* DC3000 (*AvrRpm1*)- and *Pst* DC3000 (*AvrRpt2*)-infiltrated leaves. In contrast, necrotrophic pathogens assimilate nutrients from dead tissues and thus profit from host cell death (Govrin and Levine, 2000; Mayer et al., 2011). Enhanced cell death in *CRK36OE* plants would provide favorable conditions for the proliferation of *A. brassicicola* and *E. carotovora*. Other types of cell death, e.g., cell death triggered by fungal toxin FB1 and during senescence, were also positively affected by CRK36. These results suggest that CRK36 plays a positive role in PCD processes.

### ROS production and stomatal immunity

ROS are generated during oxidizing metabolic processes in peroxisomes, mitochondria, and chloroplasts, and have long been regarded as toxic by-products that cause photooxidative damage to DNA, proteins, and lipids. However, the identification of enzyme-mediated ROS production and detoxification mechanisms has revealed the regulatory roles of ROS in cell signaling and homeostasis (Robson and Vanlerberghe, 2002; Mittler et al., 2004; Moons, 2005; Torres et al., 2006; Bhattacharjee, 2012). ROS, acting as signaling molecules, regulate various biological processes, including cell growth, development, and abiotic and biotic stress responses (Gechev et al., 2006; Pitzschke et al., 2006; Miller et al., 2010). Most notably, ROS has been implicated in plant immunity (Torres et al., 2002; Pogány et al., 2009; Mersmann et al., 2010; Yoshioka et al., 2011; Kadota et al., 2014, 2015; Li et al., 2014). ROS production, the so-called oxidative burst, is one of the earliest responses triggered during plant-pathogen interactions, and is characterized as apoplastic and biphasic. The first phase of ROS production is a rapid and transient response that occurs within minutes of PAMP recognition during PTI (Chinchilla et al., 2007; Gimenez-Ibanez et al., 2009; Daudi et al., 2012; Liu et al., 2013; Li et al., 2014; Macho and Zipfel, 2014). The second phase of ROS production is slower but stronger, and is usually associated with the establishment of HR cell death during ETI (Torres, 2010; Tsuda and Katagiri, 2010; Yoshioka et al., 2011; Caplan et al., 2015). Plasma membrane-localized NADPH oxidases, which belong to the Respiratory Burst Oxidase Homolog (RBOH) family, are responsible for apoplastic oxidative burst during both PTI and ETI (Torres et al., 2005; Tsuda and Katagiri, 2010; Kadota et al., 2015). Functional analysis of *rboh* mutants has demonstrated that RbohD is required for ROS production in response to PAMPs and avirulent pathogens (Kadota et al., 2015; Morales et al., 2016). RbohF may also partly contribute to pathogen-induced ROS production, since *rbohD rbohF* double mutants potentiated defense phenotypes, as compared to individual mutants (Yun et al., 2011; Chaouch et al., 2012; Morales et al., 2016). It has been shown that stomatal defense is central for PTI and that it depends on RbohD-induced ROS production (Kadota et al., 2014, 2015; Li et al., 2014; Arnaud and Hwang, 2015). In this study, ROS accumulated during *AvrRpm1*- and *AvrRpt2*-induced ETI and flg22-induced PTI responses, and in both responses, ROS production was further increased by *CRK36* overexpression. In *CRK36OE* plants, flg22-induced stomatal closure and resistance to *Pst* DC3000 were enhanced, both of which required RbohD/F. Conversely, ROS burst and stomatal immune responses were decreased in *crk36* plants as compared to wild type plants. These results suggest that CRK36 plays a positive role in Rboh-mediated ROS production and stomatal defense during innate immune responses.

Yeh et al. (2015) recently reported that CRK4, CRK6, and CRK36 regulate PTI and physically interact with FLS2 in protoplasts. Whereas the binding of FLS2 with BAK1 depends on PAMP flagellin or flg22 (Chinchilla et al., 2007; Lu et al., 2010; Schulze et al., 2010), FLS2 interacted with CRK36 as well as CRK4 and CRK6 in an flg22-independent manner (Yeh et al., 2015). The association of CRK36 with FLS2 was further confirmed in our study. We additionally showed that CRK36 interacts with BIK1. As BIK1 associates with PRRs (Lu et al., 2010; Zhang et al., 2010; Couto et al., 2016), CRK36 may be a component of PRR complexes. In the present study, *CRK36* was isolated as an *A. brassicicola*-responsive gene and positively regulated fungal resistance. Fungal PAMPs, including chitin, have been identified and chitin elicitor receptor kinase 1 (CERK1) functions as a chitin-responsive PRR (Nürnberger et al., 2004). BIK1 has been shown to interact with CERK1 (Zhang et al., 2010), suggesting that CRK36 may be also involved in fungal PTI. However, BAK1 does not interact with CERK1 but is engaged in the ERECTA LRR-RLK complex during necrotrophic fungal resistance (Jordá et al., 2016). It would be important to investigate whether fungal PAMPs form PRR complexes composed of known PTI components and induce common PTI signaling.

While ROS are considered important signaling molecules, it is not known how ROS are perceived in the apoplast and how they are relayed to signaling events of PTI and ETI. Recently, Kimura et al. (2017) have reviewed the role of ROS in RLK signaling and ROS-RLK crosstalk in the context of stomatal immunity. They suggest a potential role for CRKs in the regulation of ROS production, although the exact mechanism is unclear. CRKs have DUF26 domains with conserved Cys (C-X_8_-C-X_2_-C) that have been suggested as targets of redox modification, placing them as potential “ROS receptors” or as a part of the ROS sensing system (Wrzaczek et al., 2010, 2013; Bourdais et al., 2015). It was recently shown that the extracellular Cys residues are required for CRK28-induced cell death in *N. benthamiana* (Yadeta et al., 2017). In our work, *CRK36*^*C12345A*^*OE* and *CRK36*^*C6789A*^*OE* plants with Cys-to-Ala mutations were impaired in both ETI and PTI responses. Intriguingly, flg22-triggered BIK1 phosphorylation was largely abolished in *CRK36*^C12345A^*OE* and *CRK36*^C6789A^*OE* protoplasts, although Cys mutant forms CRK36^C12345A^ and CRK36^C6789A^ were still able to associate with BIK1. We demonstrate that the conserved Cys residues in the DUF26 domains are essential for CRK36 functions.

### A proposed model for CRK36 functions in immunity

We propose a model in which CRK36 functions as an ROS receptor and participates in a positive activation loop of CRK36, BIK1, NADPH oxidase, and ROS (Figure 7C). CRK36 is activated by sensing ROS through redox modification of Cys residues in the DUF26 domains. This triggers the phosphorylation and activation of BIK1. The activated BIK1 then phosphorylates RbohD, boosting ROS burst and stomatal immunity. In our model, CRK36 activation requires the initial ROS burst triggered by BIK1, which is activated upon flg22 binding to the FLS2-BAK1 complex. It will be important to examine whether and how the FLS2-BAK1 and FLS2-CRK36 complexes are connected to regulate PTI responses. Additional studies on the role of DUF26 domains in redox regulation would enhance our understanding of the structure and function of CRKs.

## Author contributions

DL and OP designed the research. DL, YK, SK, and CR performed the research. DL and OP analyzed the data and wrote the article.

### Conflict of interest statement

The authors declare that the research was conducted in the absence of any commercial or financial relationships that could be construed as a potential conflict of interest.
