# Supplementary material for: The Arabidopsis Cysteine-Rich Receptor-Like Kinase CRK36 Regulates Immunity through Interaction with the Cytoplasmic Kinase BIK1
Source: Front Plant Sci. 2017 Oct 27;8:1856. doi: 10.3389/fpls.2017.01856 (PMC5663720; doi:10.3389/fpls.2017.01856)
Supplement: Supplementary file 1 [file Image1.PDF]

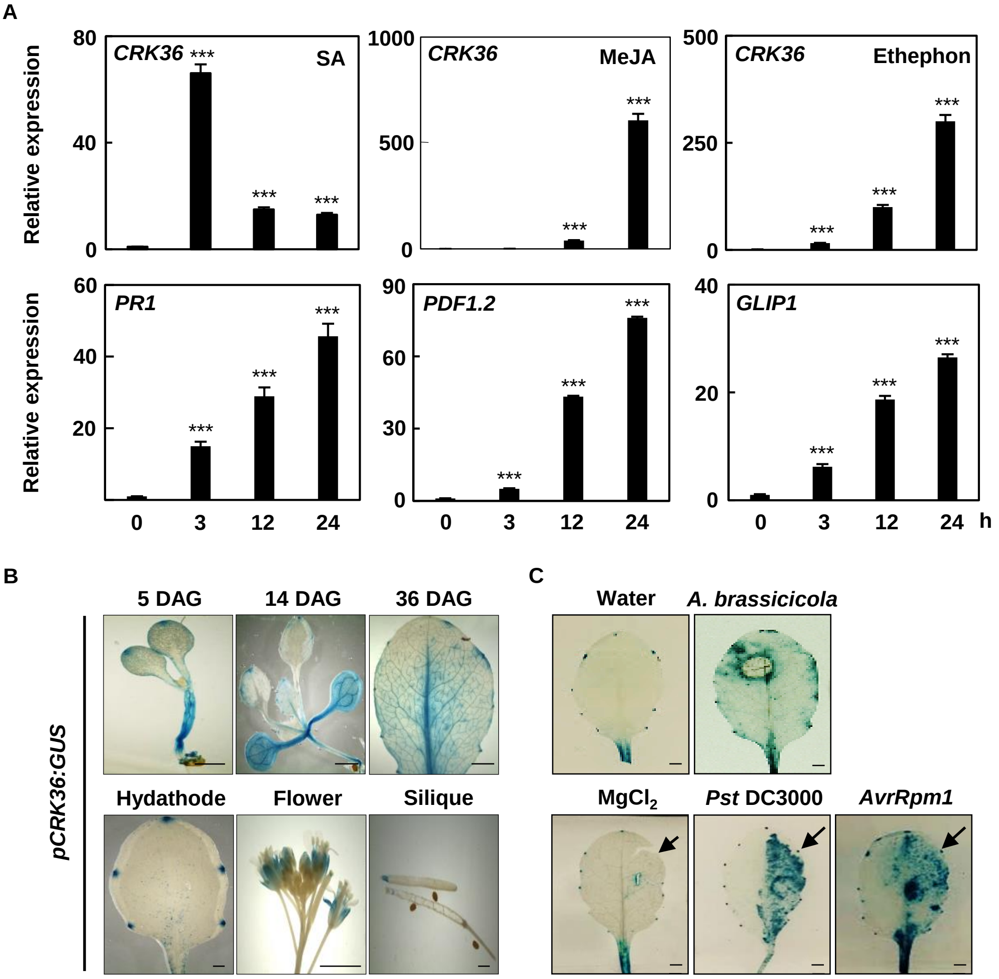

Figure S1. *CRK36* expression in response to hormone and pathogen treatments and in tissues. **(A)** *CRK36* expression in response to hormone treatments. Six-week-old plants were treated with SA (1 mM), MeJA (50 μM), and ET (1.5 mM) for the indicated times and subjected to qRT-PCR analysis. *PR1*, *PDF1.2*, and *GLIP1* were used as SA-, MeJA-, and ET-responsive marker genes, respectively. Results represent means (± SD) of 3 biological replicates. Asterisks indicate significant differences from the 0 time point (*t* test; ***$P < 0.001$). **(B)** GUS histochemical staining of *pCRK36:GUS* plants. DAG, days after germination. **(C)** GUS histochemical staining of *pCRK36:GUS* leaves infected with pathogens. Col-0 leaves were treated with water or *A. brassicicola* spores at 1 x 10^6 spores/mL for 3 days (top) and with 10 mM $MgCl_2$, *Pst* DC3000 or *Pst* DC3000 (*AvrRpm1*) at 1 x 10^6 cfu/mL for 1 day (bottom) before staining. Arrows mark infection sites. Experiments were repeated 3 times with similar results. Bars, 5 mm.
