# Supplementary material for: The Arabidopsis Cysteine-Rich Receptor-Like Kinase CRK36 Regulates Immunity through Interaction with the Cytoplasmic Kinase BIK1
Source: Front Plant Sci. 2017 Oct 27;8:1856. doi: 10.3389/fpls.2017.01856 (PMC5663720; doi:10.3389/fpls.2017.01856)
Supplement: Supplementary file 2 [file Image2.PDF]

Lee et al., Figure S2

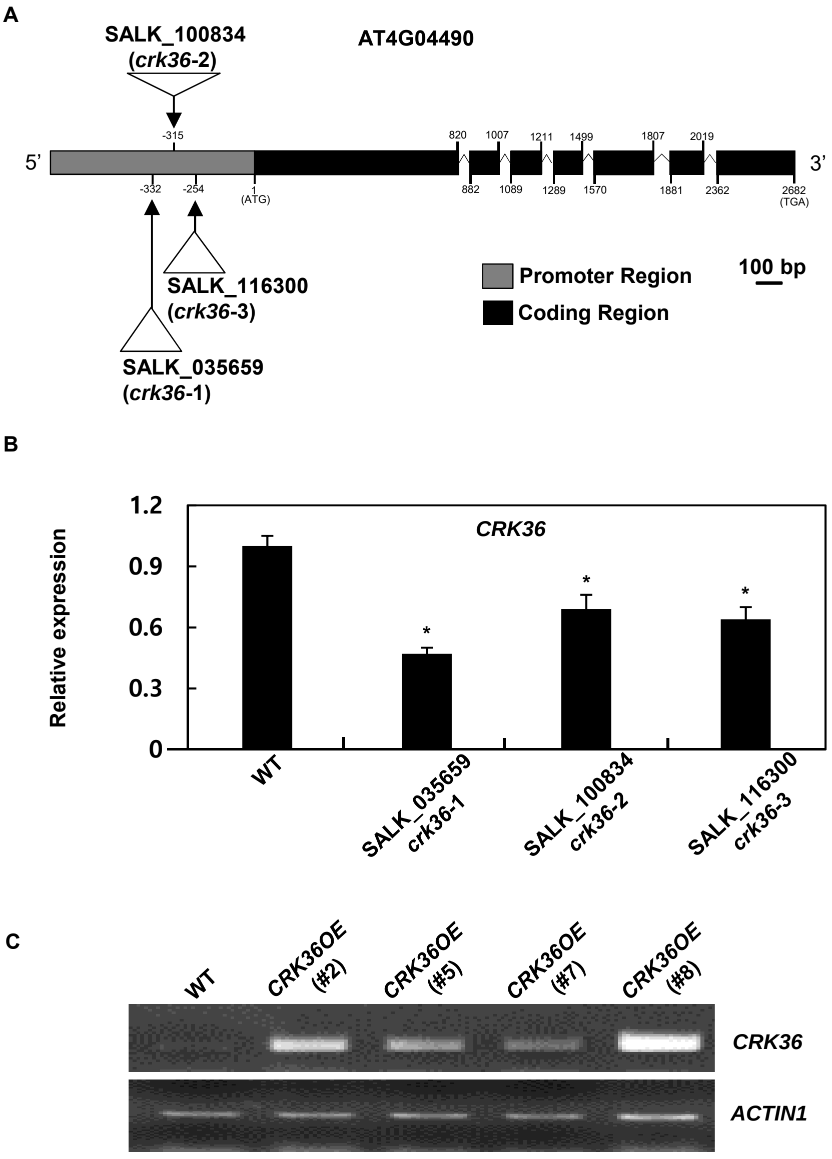

Figure S2. Preparation of *crk36* and *CRK36OE* lines. **(A)** Genomic structure of *CRK36* gene showing the positions of T-DNA insertion. gDNA sequences are represented by exons (boxes) and introns (lines). Numbers refer to nucleotides of *CRK36* genes. **(B)** qRT-PCR analysis of *CRK36* expression in T-DNA insertion *crk36* lines. Results represent means (± SD) of 3 biological replicates. Asterisks indicate significant differences from WT (*t* test; **P* < 0.05). **(C)** RT-PCR analysis of *CRK36* expression in *CRK36OE* lines. *ACTIN1* was used as a control.
