# Supplementary material for: The Arabidopsis Cysteine-Rich Receptor-Like Kinase CRK36 Regulates Immunity through Interaction with the Cytoplasmic Kinase BIK1
Source: Front Plant Sci. 2017 Oct 27;8:1856. doi: 10.3389/fpls.2017.01856 (PMC5663720; doi:10.3389/fpls.2017.01856)
Supplement: Supplementary file 3 [file Image3.PDF]

Lee et al., Figure S3

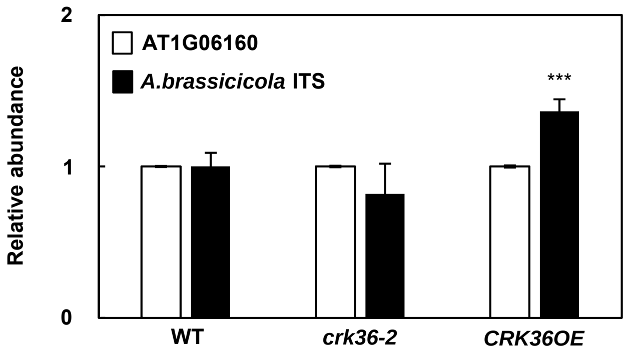

Figure S3. qPCR analysis of *A. brassicicola* ITS abundance in *crk36* and *CRK36OE* plants. Arabidopsis AT1G06160 was used as an internal control. Leaves were inoculated with fungal spore suspension (1 x 10^6 spores/mL) for 2 days. Asterisks indicate significant differences from WT (*t* test; ***P < 0.001). Experiments were repeated 3 times with similar results.
