# Supplementary material for: The Arabidopsis Cysteine-Rich Receptor-Like Kinase CRK36 Regulates Immunity through Interaction with the Cytoplasmic Kinase BIK1
Source: Front Plant Sci. 2017 Oct 27;8:1856. doi: 10.3389/fpls.2017.01856 (PMC5663720; doi:10.3389/fpls.2017.01856)
Supplement: Supplementary file 4 [file Image4.PDF]

Lee et al., Figure S4

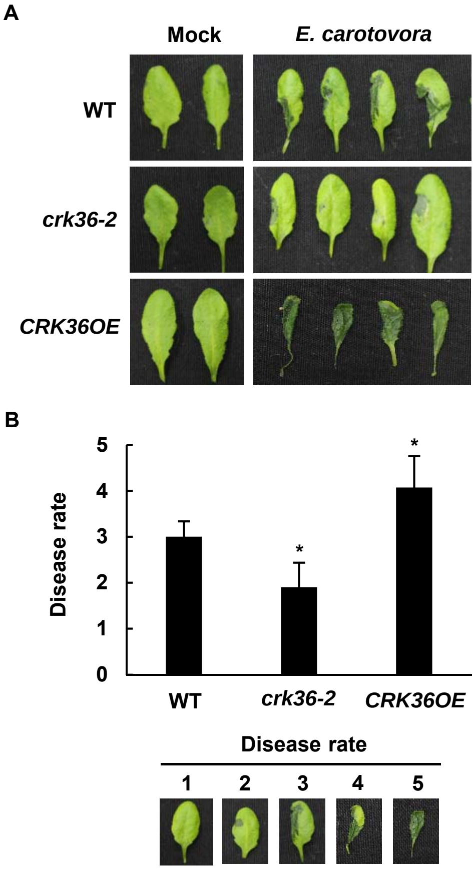

Figure S4. Responses of *crk36* and *CRK36OE* plants to *E. carotovora*. **(A)** Disease symptoms in leaves inoculated with *E. carotovora*. **(B)** Disease severity estimations (0-5) were made 2 days after pathogen inoculation (1 x 10^7 cfu/mL). Values are means ± SD (*n* = 12). Asterisks indicate significant differences from WT (*t* test; $*P < 0.05$). The experiment was repeated 3 times with similar results.
