# Supplementary material for: The Arabidopsis Cysteine-Rich Receptor-Like Kinase CRK36 Regulates Immunity through Interaction with the Cytoplasmic Kinase BIK1
Source: Front Plant Sci. 2017 Oct 27;8:1856. doi: 10.3389/fpls.2017.01856 (PMC5663720; doi:10.3389/fpls.2017.01856)
Supplement: Supplementary file 5 [file Image5.PDF]

Lee et al., Figure S5

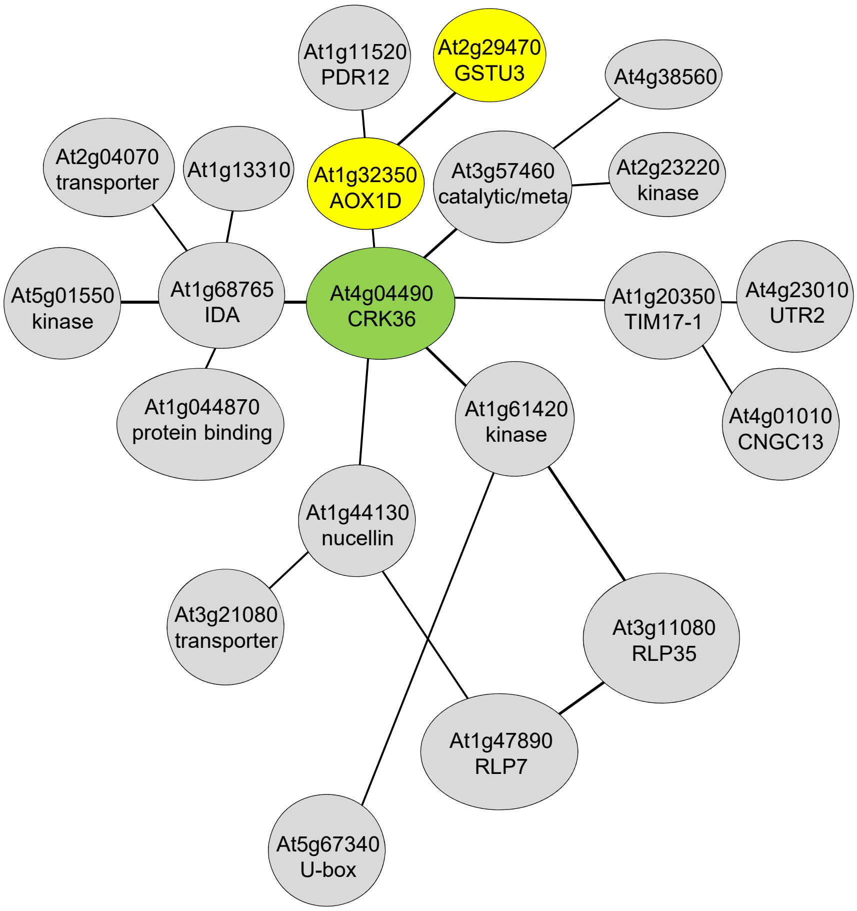

Figure S5. Coexpressed gene network for *CRK36*. Analysis of a gene coexpression database ATTED-II (http://atted.jp/) provided the list of co-expressed genes. Redox system-related genes *AOX1D* and *GSTU3* are shown in yellow. The lines indicate that two genes are co-expressed.
