# Supplementary material for: The Arabidopsis Cysteine-Rich Receptor-Like Kinase CRK36 Regulates Immunity through Interaction with the Cytoplasmic Kinase BIK1
Source: Front Plant Sci. 2017 Oct 27;8:1856. doi: 10.3389/fpls.2017.01856 (PMC5663720; doi:10.3389/fpls.2017.01856)
Supplement: Supplementary file 6 [file Image6.PDF]

# Lee et al., Figure S6

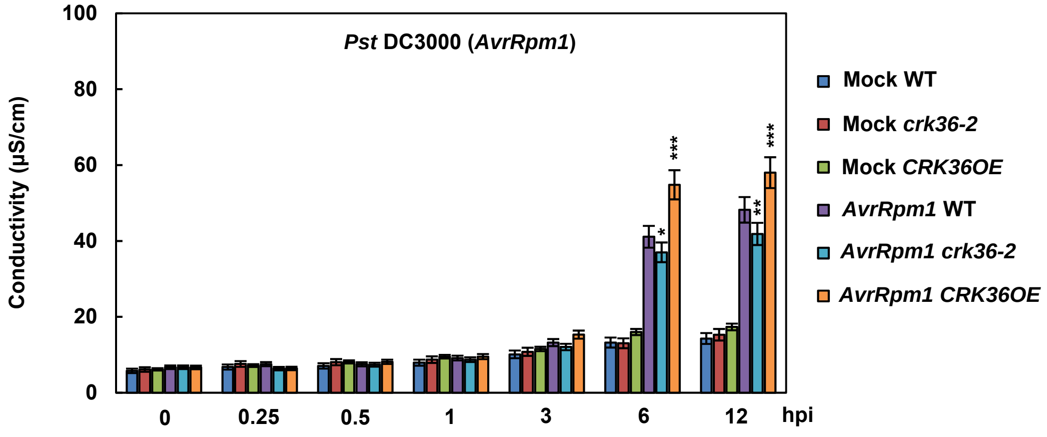


**Figure S6.** Ion leakage assay in *crk36* and *CRK36OE* plants. Leaves of 6-week-old plants were infiltrated with 10 mM $MgCl_2$ (mock) or 2 x $10^8$ cfu/mL *Pst* DC3000 (*AvrRpm1*), and conductivity was measured at the indicated times. Results represent means ± SD ($n$ = 9). Asterisks indicate significant differences from WT ($t$ test; *$P$ < 0.05; **$P$ < 0.01; ***$P$ < 0.001). Experiments were repeated 3 times with similar results. hpi, hours post-inoculation.
