# Supplementary material for: The Arabidopsis Cysteine-Rich Receptor-Like Kinase CRK36 Regulates Immunity through Interaction with the Cytoplasmic Kinase BIK1
Source: Front Plant Sci. 2017 Oct 27;8:1856. doi: 10.3389/fpls.2017.01856 (PMC5663720; doi:10.3389/fpls.2017.01856)
Supplement: Supplementary file 7 [file Image7.PDF]

Lee et al., Figure S7

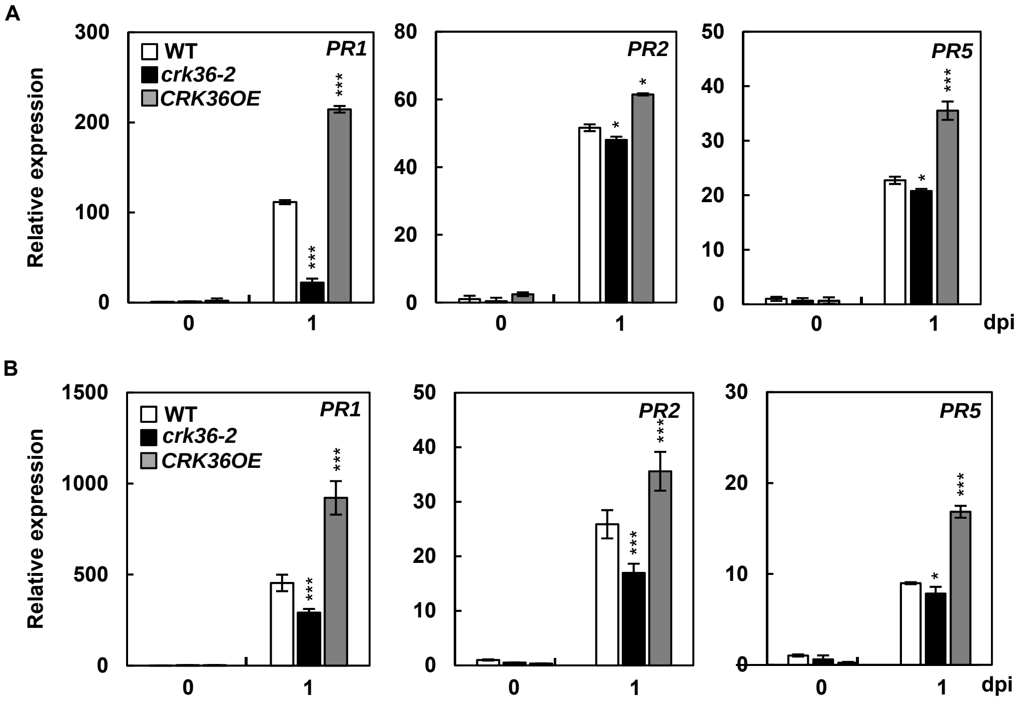

Figure S7. *PR* gene expression in *crk36* and *CRK36OE* plants. **(A)** qRT-PCR analysis of *PR* gene expression in leaves inoculated with *Pst* DC3000. **(B)** qRT-PCR analysis of *PR* gene expression in leaves inoculated with *Pst* DC3000 (*AvrRpm1*). Results represent means (± SD) of 3 biological replicates. Leaves were inoculated with *Pst* DC3000 or *Pst* DC3000 (*AvrRpm1*) at 1 x 10^6 cfu/mL for 1 day. Asterisks indicate significant differences from WT (*t* test; **P* < 0.05; ****P* < 0.001). Experiments were repeated 3 times with similar results. dpi, days post-inoculation.
