# Supplementary material for: The Arabidopsis Cysteine-Rich Receptor-Like Kinase CRK36 Regulates Immunity through Interaction with the Cytoplasmic Kinase BIK1
Source: Front Plant Sci. 2017 Oct 27;8:1856. doi: 10.3389/fpls.2017.01856 (PMC5663720; doi:10.3389/fpls.2017.01856)
Supplement: Supplementary file 8 [file Image8.PDF]

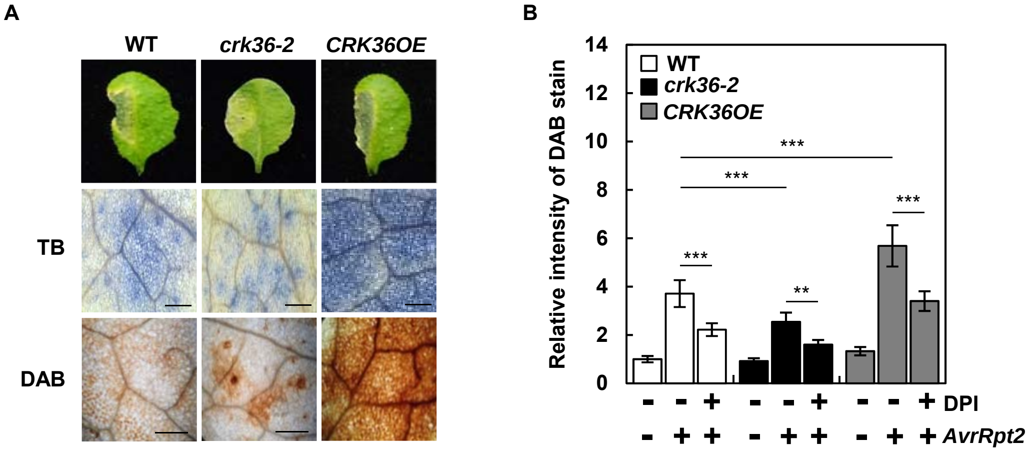

Figure S8. Cell death and ROS production in *crk36* and *CRK36OE* plants challenged with *Pst* DC3000 (*AvrRpt2*). **(A)** Phenotypes of leaves (top), cell death determined by trypan blue (TB) staining (middle), and ROS production by DAB staining (bottom) after *Pst* DC3000 (*AvrRpt2*) treatment. **(B)** Quantification of DAB-stained ROS in (a) and DPI effect on ROS production after *Pst* DC3000 (*AvrRpt2*) treatment. Leaves were pre-treated with 10 μM DPI for 1 h and then infected with pathogens. Values are means ± SD (n = 6). Leaves were inoculated with *Pst* DC3000 (*AvrRpt2*) at 1 x 10^7 cfu/mL for 1 day. Asterisks indicate significant differences from WT and between untreated and DPI-treated plants (t test; $^{**}P < 0.01$; $^{***}P < 0.001$). Experiments were repeated 3 times with similar results.
