# Supplementary material for: The Arabidopsis Cysteine-Rich Receptor-Like Kinase CRK36 Regulates Immunity through Interaction with the Cytoplasmic Kinase BIK1
Source: Front Plant Sci. 2017 Oct 27;8:1856. doi: 10.3389/fpls.2017.01856 (PMC5663720; doi:10.3389/fpls.2017.01856)
Supplement: Supplementary file 9 [file Image9.PDF]

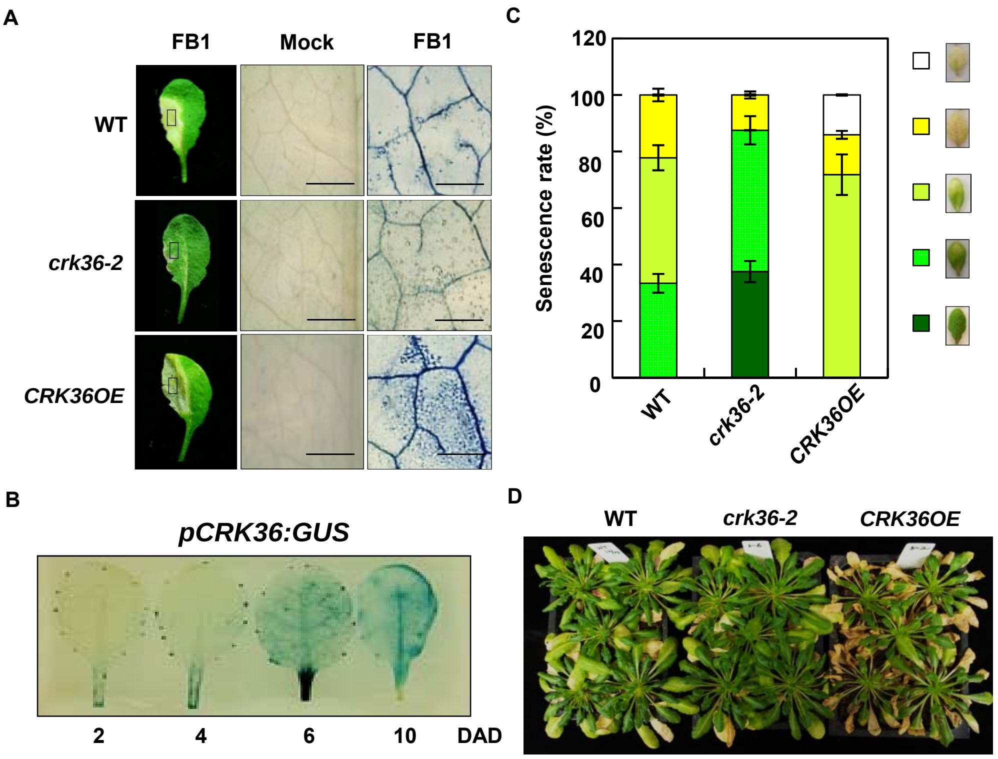

Figure S9. Cell death in fumonisin B1-treated and senescing leaves of *crk36* and *CRK36OE* plants. **(A)** Phenotypes of leaves (left) and cell death stained with trypan blue (middle and right). Enlarged images (right) of the boxed areas (left) are shown. Leaves were infiltrated with 10% DMSO (mock) or 10 µM fumonisin B1 (FB1) for 6 days. Bars, 500 µm. **(B)** GUS activity in *pCRK36:GUS* leaves during dark-induced senescence. DAD, days after dark treatment. **(C)** Dark-induced leaf senescence. Leaves were kept in the dark for 7 days and divided into 5 senescence levels according to leaf color. Results represent means ± SD (n = 24). **(D)** Natural leaf senescence. Ten-week-old plants grown under short-day conditions were photographed. Experiments were repeated 3 times with similar results.
