# Supplementary material for: The Arabidopsis Cysteine-Rich Receptor-Like Kinase CRK36 Regulates Immunity through Interaction with the Cytoplasmic Kinase BIK1
Source: Front Plant Sci. 2017 Oct 27;8:1856. doi: 10.3389/fpls.2017.01856 (PMC5663720; doi:10.3389/fpls.2017.01856)
Supplement: Supplementary file 10 [file Image10.PDF]

Lee et al., Figure S10

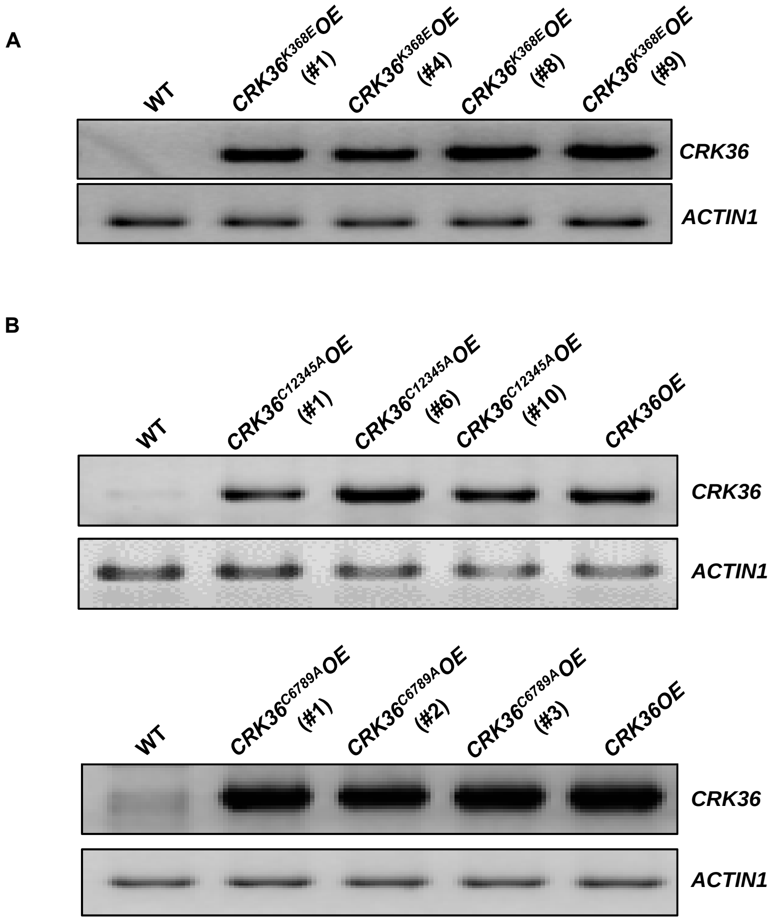

Figure S10. Generation of kinase-dead and Cys-mutated *CRK36* transgenic plants. **(A)** RT-PCR analysis of *CRK36* expression in *CRK36*^{K368E}*OE* lines. **(B)** RT-PCR analysis of *CRK36* expression in *CRK36*^{C12345A}*OE* (top) and *CRK36*^{C6789A}*OE* (bottom) lines. *ACTIN1* was used as a control.
