# Supplementary material for: The Arabidopsis Cysteine-Rich Receptor-Like Kinase CRK36 Regulates Immunity through Interaction with the Cytoplasmic Kinase BIK1
Source: Front Plant Sci. 2017 Oct 27;8:1856. doi: 10.3389/fpls.2017.01856 (PMC5663720; doi:10.3389/fpls.2017.01856)
Supplement: Supplementary file 11 [file Image11.PDF]

# Lee et al., Figure S11

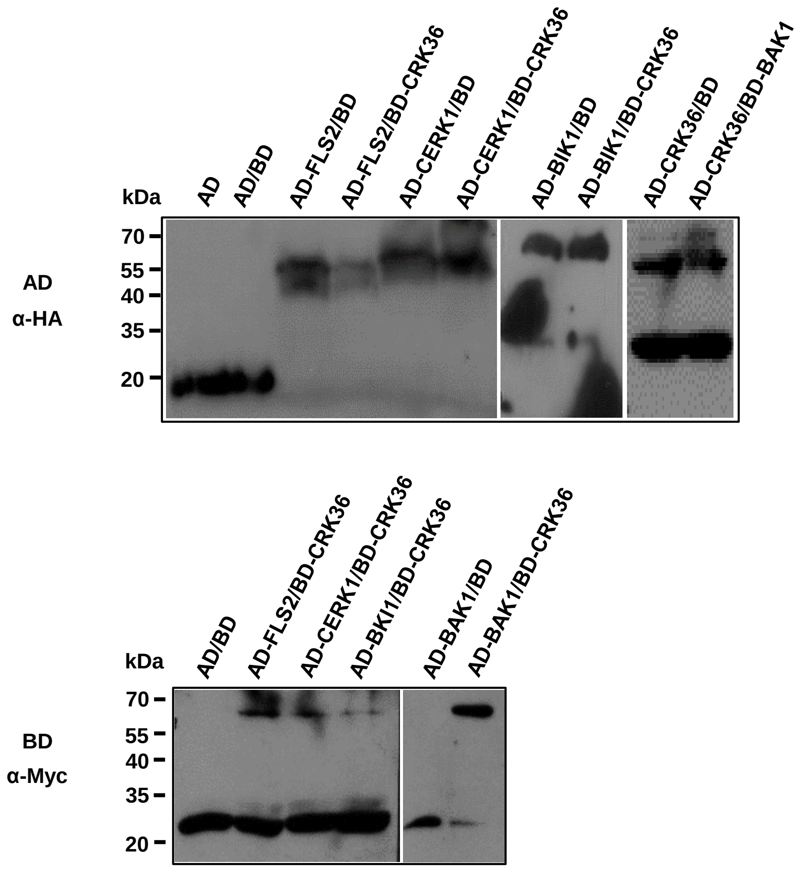


**Figure S11.** Validation of protein expression in transfected yeast cells. Protein extracts were prepared from yeast cells transfected with the indicated constructs. Proteins fused with GAL4 activation domain (AD) and DNA binding domain (BD) were detected by western blotting (WB) with anti-HA (top) and anti-Myc (bottom) antibodies, respectively.
