# Supplementary material for: The Arabidopsis Cysteine-Rich Receptor-Like Kinase CRK36 Regulates Immunity through Interaction with the Cytoplasmic Kinase BIK1
Source: Front Plant Sci. 2017 Oct 27;8:1856. doi: 10.3389/fpls.2017.01856 (PMC5663720; doi:10.3389/fpls.2017.01856)
Supplement: Supplementary file 12 [file Image12.PDF]

Lee et al., Figure S12

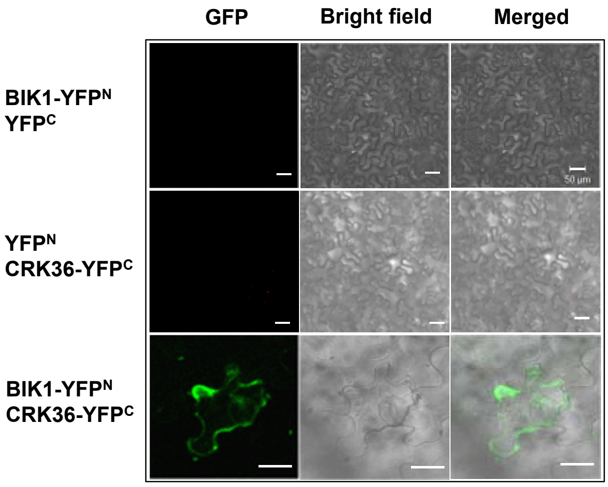

Figure S12. BiFC assay for interaction between CRK36 and BIK1 in *N. benthamiana*. YFP^N, YFP^C, BIK1-YFP^N, and CRK36-YFP^C were co-expressed in *N. benthamiana* leaves for 24 h as indicated. Reconstituted YFP fluorescence was visualized under a confocal microscope. Bars, 50 µm.
