# Supplementary material for: The Arabidopsis Cysteine-Rich Receptor-Like Kinase CRK36 Regulates Immunity through Interaction with the Cytoplasmic Kinase BIK1
Source: Front Plant Sci. 2017 Oct 27;8:1856. doi: 10.3389/fpls.2017.01856 (PMC5663720; doi:10.3389/fpls.2017.01856)
Supplement: Supplementary file 13 [file Table1.PDF]

Table S1. List of primers used for PCR, qPCR, and qRT-PCR

Gene	Upstream primer	Downstream primer	Comment
ACTIN1	5'-GGTGTCATGGTTGGTATGGGTC-3'	5'-CCTCTGTGAGTAGAACTGGGTGC-3'	RT-PCR
ELF1A	5'-GGCAAGGAGAAGGTACACAT-3'	5'-CAATCACACGCTTGTCAATA-3'	qRT-PCR
PR1	5'-TCGTCTTTGTAGCTCTTGTAGGTG-3'	5'-TAGATTCTCGTAATCTCAGCTCT-3'	qRT-PCR
PR2	5'-CGTTGTGGCTCTTTACAAACAACAAAAC-3'	5'-CAAATTAACTTCATACTTAGACTGTCGAT-3'	qRT-PCR
PR5	5'-ATGGCAAATATCTCCAGTATTCACA-3'	5'-ATGTCGGCGCAAGCCGCGTAC-3'	qRT-PCR
PDF1.2	5'-GCTAAGTTTGCTTCCATCATCACC-3'	5'- AACATGGGACGTAACAGATACACAC-3'	qRT-PCR
GLIP1	5'-GGTTTGAGACGGCTAAATC-3'	5'-GTTCAAACAGCGCTTTGAG-3'	qRT-PCR
FRK1	5'-ATCTTCGCTTGGAGCTTCTC-3'	5'-TGCAGCGCAAGGACTAGAG-3'	qRT-PCR
WRKY29	5'-CTCCATACCCAAGGAGTTATTACAG-3'	5'-CGGGTTGGTAGTTCATGATTG-3'	qRT-PCR
AOX1D	5'-CTTTCACAACCCAAATGGTACG-3'	5'-TTGTCACATAATCTTTAATGATATCC-3'	qRT-PCR
GSTU3	5'-GCTTATAGGGTCTTGGGCAAGCCC-3'	5'-CCATACCTTCCCAAGCCCGAGC-3'	qRT-PCR
CRK36	5'-CCGGAGTACGTGAGACATGG-3'	5'-TCACCGAGGATACAAGACTGTAATC-3'	RT-PCR
	5'-AAGAGAATGCAGCAAAGAGACCAACC-3'	5'-GGAATATTCACCGAGGATACAAGACTG-3'	qRT-PCR
	5'-ATGGAAAGATCCAATCTTTTCC-3'	5'-ATGAGGAGTTTCAGATCATCCA-3'	full-length
	5'-GGGGTCTAGATATCTCAGTCATTGTCAATATCGG-3'	5'-GGGGCCATGGTTATCTATTAACGTTTCCTTATTAAA-3'	promoter
	5'-GGGGTCTAGAATGGAAAGATCCAATCTTT-3'	5'-TTTTGGATCCCCGAGGATACAAGACTGT-3'	BiFC
	5'-AAAACATATGTATCGCCGGATGCGGAGG-3'	5'-TTTTGGATCCCCGAGGATACAAGACTGT-3'	Y2H
BIK1	5'-AACATATGGGTTCTTGCTTCAGTTCTC-3'	5'-TTGGATCCCTACACAAGGTGCCTGCCAA-3'	Y2H
	5'-GGGTCTAGAATGGGTTCTTGCTTCAGTTC-3'	5'-TTTGGATCCCACAAGGTGCCTGCCAAAAG-3'	BiFC
	5'-AAAGGATCCATGGGTTCTTGCTTCAGTTC-3'	5'-AAACTCGAGCTACACAAGGTGCCTGCCAA-3'	GST
	5'-TTTGGATCCCACAAGGTGCCTGCCAAAAG-3'	5'-TTTAGGCCTCACAAGGTGCCTGCCAAAG-3'	HA
FLS2	5'-AACATATGTGTTGCAAGAAAAAAGAAAAAA-3'	5'-TTGGATCCTAAACTTCTCGATCCTCGTTAC-3'	Y2H
CERK1	5'-GGGCATATGTATGCTTACCGGAAGAATAAGT-3'	5'-TTGGATCCCTACCGGCCGGACATAAGAC-3'	Y2H
BAK1	5'-TTCATATGCGAAGGAAAAAGCCGCAGGA-3'	5'-TTGGATCCTCTTGGACCCGAGGGGTATT-3'	Y2H
CRK36^{mut}	5'-CTCTTGCCCTAGCTCGAAAACACTAC-3'	5'-GTAGTGTTTTCGAGCTAGGGCAAGAG-3'	C1A
	5'-CAAGCTGCTCGTCGCGCCGTCGATAG-3'	5'-CTATCGACGGCGCGACGAGCAGCTTG-3'	C23A
	5'-CTGTTAACACAAGCCCGAGGCAAAAC-3'	5'-GTTTTGCCTCGGGCTTGTGTTAACAG-3'	C4A
	5'-GCGTACTAGAGCAGAAACGTTTGC-3'	5'-GCGTACTAGAGCAGAAACGTTTGC-3'	C5A
	5'-GTTAATATGTTGATGCAAGCCACACCTGA-3'	5'-TCAGGTGTGGCTTGCATCAACATATTAAC-3'	C6A
	5'-CCAGTGACGCTAATCATGCTCTGAGAGA-3'	5'-TCTCTCAGAGCATGATTAGCGTCACTGG-3'	C78A
	5'-GCCCGTCCTAGTGCTTATTTCCGATGGG-3'	5'-CCCATCGGAAATAAGCACTAGGACGGGC-3'	C9A
	5'-GATAGCGGTAGAGAGATTAGC-3'	5'-GCTAATCTCTCTACCGCTATC-3'	K368E
AT1G06160	5'-GCCCTACTGATAACTAC-3'	5'-GCGTCATAACAACACTC-3'	qPCR
A. brassicicola ITS	5'-TCTCCAGTTTGCTGGAGACT-3'	5'-GGATGCTGACCTTGGCTGGA-3'	qPCR
