# Supplementary material for: The Arabidopsis Cysteine-Rich Receptor-Like Kinase CRK36 Regulates Immunity through Interaction with the Cytoplasmic Kinase BIK1
Source: Front Plant Sci. 2017 Oct 27;8:1856. doi: 10.3389/fpls.2017.01856 (PMC5663720; doi:10.3389/fpls.2017.01856)
Supplement: Supplementary file 15 [file Table3.PDF]

**Table S3.** Differentially expressed genes encoding kinases 6 h after *A. brassicicola* inoculation

| Locus | Gene Symbol | Description | Fold Change | *P*-Value |
|---|---|---|---|---|
| AT4G04490 | *CRK36* | Cysteine-rich receptor-like protein kinase | 2.04 | 2.44E-04 |
| AT1G51800 | *IOS1* | Leucine-rich repeat protein kinase | 2.28 | 2.44E-04 |
| AT5G51830 | | pfkB-like carbohydrate kinase | 2.56 | 2.44E-04 |
| AT3G45330 | *LECRK-I.1* | Lectin receptor kinase | 2.45 | 2.44E-04 |
| AT4G04700 | *CPK27* | Calcium dependent kinase | 2.56 | 2.44E-04 |
| AT1G78940 | *WAK1* | Wall-associated kinase-like | 3.29 | 6.36E-04 |
| AT3G25250 | *AGC2* | cGMP-dependent and protein kinase C | 3.40 | 6.06E-04 |
| AT2G30040 | *MAPKKK14* | MAP kinase kinase kinase | 0.31 | 5.86E-04 |
| AT1G73500 | *ATMKK9* | MAP kinase kinase | 0.34 | 7.32E-04 |
| AT4G00340 | *RLK4* | Receptor-like protein kinase | 0.46 | 3.76E-04 |
